# Cathepsin K cleavage of angiopoietin-2 creates detrimental Tie2 antagonist fragments in sepsis

**DOI:** 10.1172/JCI174135

**Published:** 2025-03-03

**Authors:** Takashi Suzuki, Erik Loyde, Sara Chen, Valerie Etzrodt, Temitayo O. Idowu, Amanda J. Clark, Marie Christelle Saade, Brenda Mendoza Flores, Shulin Lu, Gabriel Birrane, Vamsidhara Vemireddy, Benjamin Seeliger, Sascha David, Samir M. Parikh

**Affiliations:** 1 Division of Nephrology, Department of Medicine, UT Southwestern Medical Center, Dallas, Texas, USA.; 2 Center for Vascular Biology Research, Beth Israel Deaconess Medical Center and Harvard Medical School, Boston, Massachusetts, USA.; 3 Division of Pediatric Nephrology, Department of Pediatrics, UT Southwestern Medical Center and Children’s Medical Center, Dallas, Texas, USA.; 4 Division of Experimental Medicine, Department of Medicine, Beth Israel Deaconess Medical Center and Harvard Medical School, Boston, Massachusetts, USA.; 5 Department of Respiratory Medicine, Hannover Medical School, Hannover, Germany.; 6 Biomedical Research in End-Stage and Obstructive Lung Disease, Hannover Medical School, German Center for Lung Research, Hannover, Germany.; 7 Institute of Intensive Care Medicine, University Hospital Zurich, Zurich, Switzerland.; 8 Department of Pharmacology, UT Southwestern Medical Center, Dallas, Texas, USA.

**Keywords:** Inflammation, Vascular biology, Endothelial cells

## Abstract

Elevated angiopoietin-2 is associated with diverse inflammatory conditions, including sepsis, a leading global cause of mortality. During inflammation, angiopoietin-2 antagonizes the endothelium-enriched receptor Tie2 to destabilize the vasculature. In other contexts, angiopoietin-2 stimulates Tie2. The basis for context-dependent antagonism remains incompletely understood. Here, we show that inflammation-induced proteolytic cleavage of angiopoietin-2 converts this ligand from Tie2 agonist to antagonist. Conditioned media from stimulated macrophages induced endothelial angiopoietin-2 secretion. Unexpectedly, this was associated with reduction of the 75 kDa full-length protein and appearance of new 25 and 50 kDa C-terminal fragments. Peptide sequencing proposed cathepsin K as a candidate protease. Cathepsin K was necessary and sufficient to cleave angiopoietin-2. Recombinant 25 and 50 kDa angiopoietin-2 fragments (cANGPT2_25_ and cANGPT2_50_) bound and antagonized Tie2. Cathepsin K inhibition with the phase 3 small-molecule inhibitor odanacatib improved survival in distinct murine sepsis models. Full-length angiopoietin-2 enhanced survival in endotoxemic mice administered odanacatib and, conversely, increased mortality in the drug’s absence. Odanacatib’s benefit was reversed by heterologous cANGPT2_25_. Septic humans accumulated circulating angiopoietin-2 fragments, which were associated with adverse outcomes. These results identify cathepsin K as a candidate marker of sepsis and a proteolytic mechanism for the conversion of angiopoietin-2 from Tie2 agonist to antagonist, with therapeutic implications for inflammatory conditions associated with angiopoietin-2 induction.

## Abstract 2

Sepsis-induced cleavage of Angiopoietin-2 converted it from a Tie2 agonist to an antagonist through cathepsin K-mediated proteolysis. Inhibiting cathepsin K with odanacatib improved survival in sepsis, highlighting its potential as a therapeutic target.

## Graphical abstract

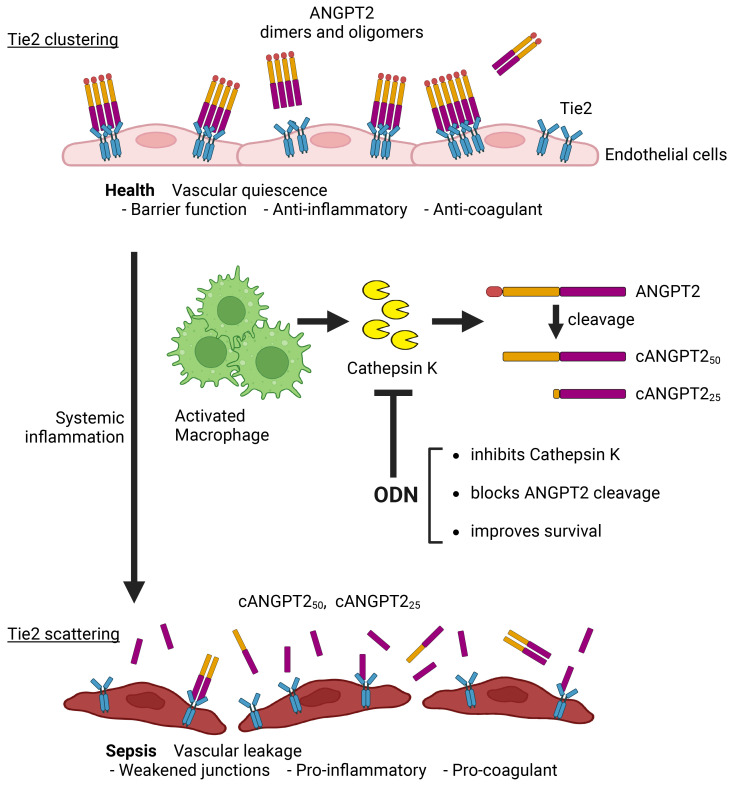

## Introduction

Of all acute illnesses, the public health burden of sepsis may be the most pressing (1–3) — patients spend more time in intensive care units (ICUs) and hospitals and more frequently suffer long-term health impairments compared with patients with any other admission diagnosis (1). The vascular response to severe infection is progressive, with worsening hyperpermeability, procoagulant response, and increased adhesiveness to leukocytes (4–6). Work from many groups has proposed that deactivation of homeostatic Tie2 signaling in the vascular endothelium regulates a dramatic phenotypic shift in microvessels that contributes to organ edema, circulatory shock, coagulopathy, and, ultimately, death (7–14).

Regulation of Tie2 is finely tuned to maintain vascular homeostasis. Phosphorylated Tie2 maintains quiescence by blunting the expression of surface adhesion and procoagulant molecules as well as fortifying microvascular barrier function. Two highly homologous ligands, angiopoietin-1 (ANGPT1) and angiopoietin-2 (ANGPT2), compete for the same binding site on Tie2 (15–17). ANGPT1 is a canonical agonist; ANGPT2 exerts a context-dependent action. During development and angiogenesis, ANGPT2 stimulates Tie2 (18–20). However, during inflammation, ANGPT2 antagonizes Tie2 (7, 11, 21–25). Several mechanisms have been identified to account for the context specificity of ANGPT2: an autocrine or intracrine binding of Tie2 at endothelial cells (ECs) (26); regulation of ANGPT2-dependent Tie2 signaling by the paralog receptor Tie1 (13, 27, 28); regulation of ANGPT2-dependent Tie2 signaling by the transmembrane tyrosine phosphatase vascular endothelial protein tyrosine phosphatase (VE-PTP) (18); and specific receptor-binding residues that differ between the ANGPT proteins (29).

The ANGPT N-terminus contains superclustering (SCD) and coiled-coil domains (CC), which are responsible for multimerization, and a C-terminus whose fibrinogen-like (FL) domain binds Tie2 (17, 30, 31). Native ANGPT1 exists as a tetramer and higher-order oligomers, whereas ANGPT2 is less multimerized (32, 33). This difference in quaternary structure accounts for the differential activity of ANGPT1 versus ANGPT2 at Tie2, with stoichiometry studies proposing that ANGPT1 efficiently clusters Tie2 monomers at the EC surface to promote receptor phosphorylation, while ANGPT2 ligation prevents ANGPT1 from binding Tie2 and instead scatters Tie2 molecules (32, 33).

Here, we show that inflammation-induced upregulation of ANGPT2 lead to extracellular cleavage of full-length ANGPT2 to generate a specific 25 kDa C-terminal fragment that was unable to oligomerize and instead acquired inhibitory activity against ANGPT1-induced Tie2 signaling. The responsible protease, cathepsin K (CATK), was released from activated macrophages. Pharmacological CATK inhibition improved survival in distinct models of sepsis. CATK activity determined whether excess full-length ANGPT2 improves or exacerbates sepsis outcomes. The benefit of CATK inhibition was overcome by expression of the 25 kDa fragment (cANGPT2_25_) and attenuated by genetic depletion of ANGPT2. Finally, abundance of the cleavage product in sera from critically ill humans associates with adverse organ-specific and global outcomes.

## Results

### Activated macrophage conditioned media processes ANGPT2 into distinct fragments.

**Figure 1:**
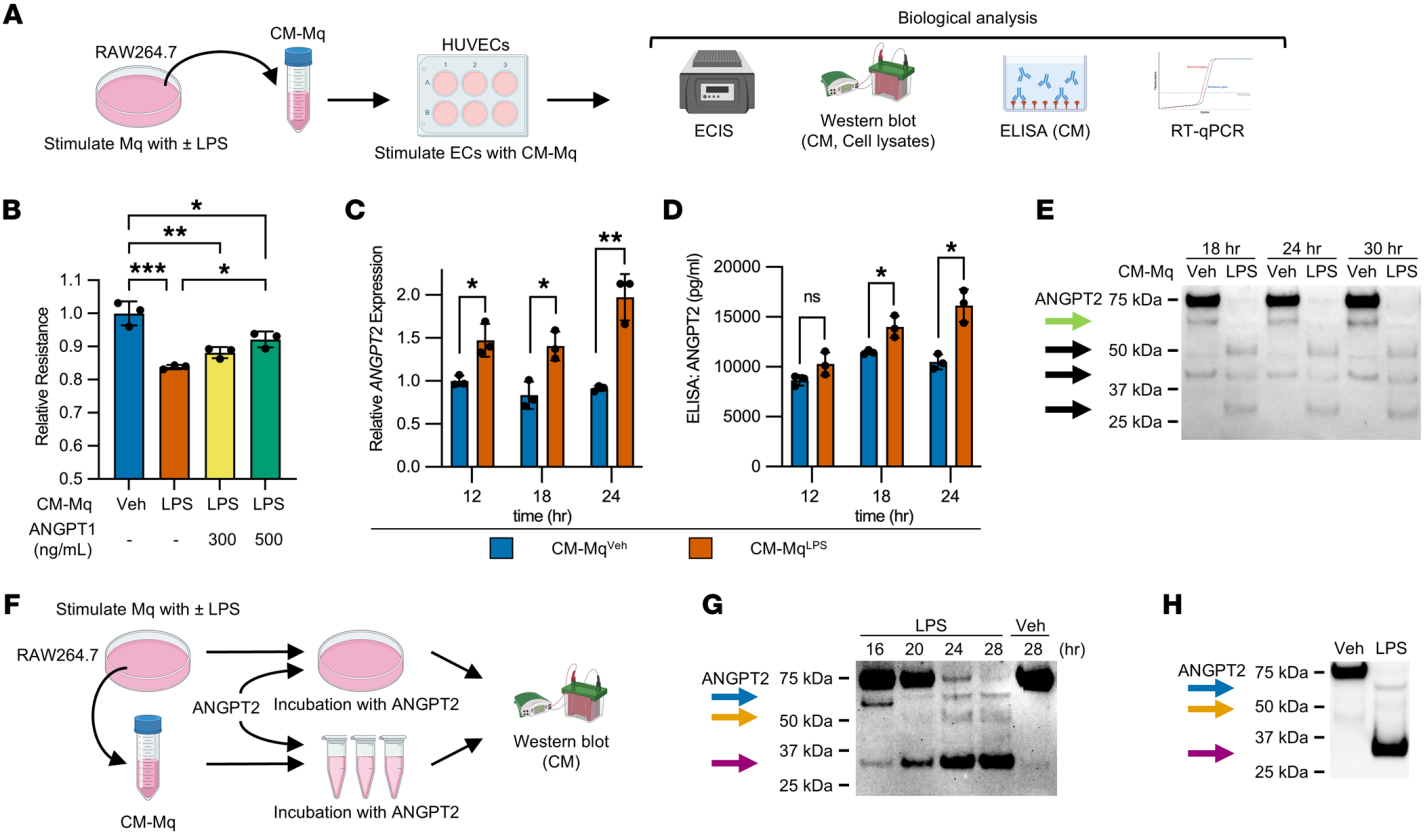
Conditioned media from activated macrophages processes ANGPT2 into distinct fragments. (**A**) Schematic representation of experiments evaluating the effects of macrophages’ conditioned media (CM-Mq) on HUVECs. ECIS, electric cell-substrate impedance sensing. (**B**) Transendothelial electrical resistance in confluent HUVEC monolayers treated with CM-Mq for 20 hours (*n* = 3 per group, ANOVA with multiple comparison *P* values; individual resistance curves in Supplemental Figure 1A). (**C**–**E**) HUVECs stimulated with CM-Mq^Veh^ or CM-Mq^LPS^ for the indicated time. *ANGPT2* mRNA levels measured by (**C**) RT-qPCR on HUVEC homogenates, and ANGPT2 protein level in CM measured by (**D**) ELISA or (**E**) Western analysis (*n* = 3 per group, respectively). Black arrows to putative processed versions of ANGPT2. The green arrow points to putative ANGPT2_443_ isoform. An uncropped image, including 0-hour stimulation with CM-Mq^Veh^ or CM-Mq^LPS^, is shown in Supplemental Figure 1E. (**F**) Schematic representation of experiments to test if CM-Mq^LPS^ induces cell-free posttranslational modification on ANGPT2. (**G**) RAW264.7 cells were treated with LPS and concomitant application of recombinant ANGPT2 for the indicated time, after which ANGPT2 in CM was analyzed by Western blotting. Three arrowheads (blue, yellow, and purple) indicate putative processed versions of ANGPT2. (**H**) Incubation of recombinant ANGPT2 with CM-Mq^LPS^ in the absence of cells for 37°C for 24 hours prior to Western blot analysis. Western analysis images are representative of at least 3 independent experiments. *P < 0.05, **P < 0.01, ***P < 0.001 by Mann-Whitney except as noted.

To evaluate the effects of activated macrophages on Tie2 signaling in ECs, we stimulated HUVECs with conditioned media (CM) from murine macrophage-like RAW264.7 cells stimulated with Gram-negative bacterial LPS (CM-Mq^LPS^, Figure 1A). CM-Mq^LPS^ suppressed endothelial monolayer barrier function compared with CM from vehicle-stimulated RAW264.7 cells (CM-Mq^Veh^, Figure 1B, and Supplemental Figure 1A; supplemental material available online with this article; https://doi.org/10.1172/JCI174135DS1). This downregulation was recovered proportionally by concomitant application of ANGPT1, suggesting that diminished Tie2 signaling following CM-Mq^LPS^ application contributes to endothelial barrier dysfunction induced by CM-Mq^LPS^. Because CM-Mq^LPS^ did not alter total Tie2 levels in HUVECs (Supplemental Figure 1C), we hypothesized that CM-Mq^LPS^ affected Tie2 signaling by changing ANGPT2. As expected, CM-Mq^LPS^ induced upregulation of *ANGPT2* mRNA without altering intracellularly retained ANGPT2 in HUVECs (Figure 1C and Supplemental Figure 1B). ELISA showed the expected elevated secretion of ANGPT2 from HUVECs in response to LPS (Figure 1D). Unexpectedly, however, Western analysis showed reduced full-length ANGPT2 protein accompanied by the appearance of multiple lower-molecular-weight bands in CM of HUVECs (Figure 1E and Supplemental Figure 1E). These results indicated that CM-Mq^LPS^ upregulated production and secretion of ANGPT2 from HUVECs and that CM-Mq^LPS^ also induced modification of ANGPT2 protein.

We next stimulated RAW264.7 cells with LPS and concomitant recombinant human ANGPT2 protein (Figure 1F). Western analysis of ANGPT2 revealed the appearance of 3 bands with approximate sizes of 70 kDa, 50 kDa, and 25 kDa. The approximately 25 kDa band was dominant over time, while the 75 kDa full-length ANGPT2 band progressively diminished and then was nearly undetectable (Figure 1G). The intermediate-sized bands of approximately 70 kDa and approximately 50 kDa were low in intensity, and the approximately 50 kDa band appeared smudged. Next, we incubated CM-Mq^LPS^ with recombinant human ANGPT2 at 37°C for 24 hours in the absence of any cells. This still yielded 3 bands of approximately 70 kDa, 50 kDa, and 25 kDa (Figure 1H). We tested if ANGPT2 was directly modified by ECs. HUVECs activated by phorbol ester (PMA) were stimulated with vehicle or LPS and then incubated with recombinant ANGPT2 protein for various times. We were unable to detect modified ANGPT2 bands in the CM of these HUVECs (Supplemental Figure 1D).

These results indicated that the 3 putative ANGPT2 proteins of approximately 70 kDa, 50 kDa, and 25 kDa were neither synthesized de novo nor secreted from stimulated RAW264.7 cells and that endothelium-derived ANGPT2 was processed by an activity released from stimulated RAW264.7 cells that resulted in apparent fragments.

### CATK is responsible for cleavage of ANGPT2.

**Figure 2:**
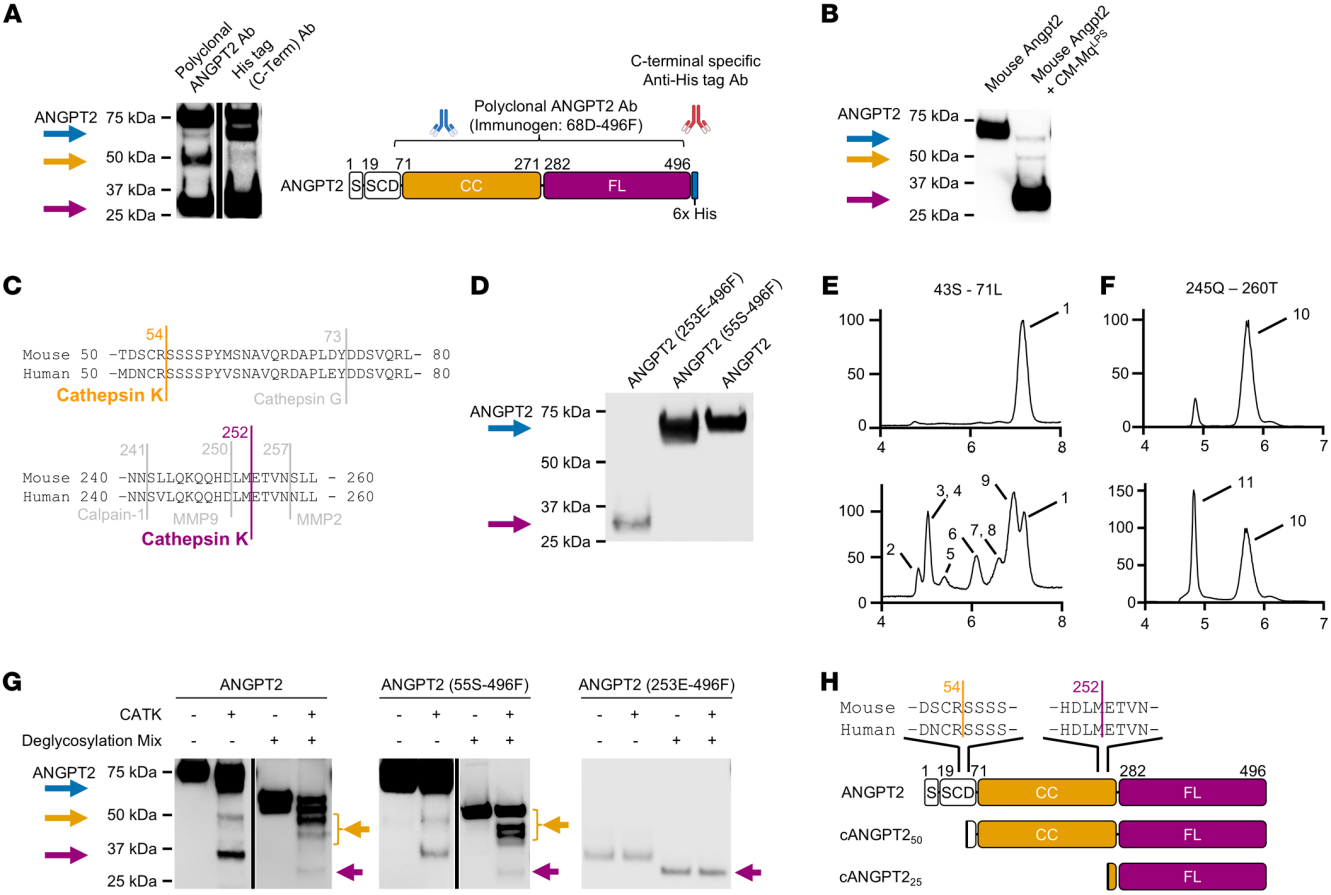
Mapping of ANGPT2 cleavage sites reveals cathepsin K–sensitive sequences. (**A**) Comparison of polyclonal ANGPT2 antibody versus monoclonal C-terminal–specific His-tag antibody in the detection of bands of ANGPT2 incubated with CM-Mq^LPS^ for 24 hours. (**B**) Incubation of murine Angpt2 with CM-Mq^LPS^ at 37°C for 24 hours before Western analysis. (**C**) In silico analysis of murine and human ANGPT2 with indicated predicted cleavage sites between amino acids 50 and 80 and between amino acids 240 and 260. (**D**) Western analysis of condition media from HEK293 cells that were transfected with full-length ANGPT2 and partial ANGPT2s (55S–496F and 253E–496F). (**E** and **F**) Mass spectrometry analysis of 2 ANGPT2 polypeptides: 42S–71L (**E**) and 245Q–260T (**F**) incubated in the presence (bottom) or absence (top) of recombinant CATK. The peaks were identified as (peak 1) SYTFLLPEMDNCRSSSSPYVSNAVQRDAPL (3,348 Da, amino acids 42S–71L), (peak 2) SYTFLLPEMDNC (1,433 Da), (peak 3) SSSSPYVSNAVQRDAPL (1,778 Da), (peak 4) MDNCRSSSSPYVSNAVQRDAPL (2,397 Da), (peak 5) LPEMDNCRSSSSPYVSNAVQRDAPL (2,737 Da), (peak 6) SYTFLLPE (969 Da), (peak 7) SYTFLLPEMDNCR (1,589 Da), (peak 8) FLLPEMDNCRSSSSPYVSNAVQRDAPL (2,997 Da), (peak 9) SYTFLLPEMDNCRSSSSPYVSNAVQ (2,796 Da), (peak 10) QKQQHDLMETVNNLLT (1,912 Da, amino acids 245Q–260T), and (peak 11) ETVNNLLT (903 Da). The peak at 4.9 minutes in the top of **F** is presumably a product from incomplete synthesis that is present in the absence of CATK (1,956 Da). Recombinant CATK cleaved ANGPT2 polypeptides at the following ANGPT2 amino acids: 44T, 46L, 49E, 53C, 54R, 66Q, and 252M. (**G**) Western analysis of ANGPT2 (full length) and partial ANGPT2s (55S–496F and 253E–496F) treated with CATK and Deglycosylation Mix (NEB) enzymes. Left arrows (yellow, purple) indicated deglycosylated cleaved ANGPT2 proteins. (**H**) Schematic protein structures of ANGPT2 and 2 cleaved ANGPT2s, cANGPT2_50_ and cANGPT2_25_. Predicted CATK cleavage sites were located at 54R and 252M. Western analysis images are representative of at least 3 independent experiments.

A polyclonal anti–C-terminal ANGPT2 antibody demonstrated apparent ANGPT2 bands, with sizes of approximately 70 kDa, 50 kDa, and 25 kDa in Western analysis (Figure 1 and Figure 2A). Detection of a C-terminal His-tag verified preservation of the C-terminus. CM-Mq^LPS^ processed murine Angpt2 in a similar manner (Figure 2B). This result indicated that cleavage sites in the ANGPT2 protein may be preserved in mice and humans.

An initial in silico analysis predicted cognate proteolytic sites that produce 3 C-terminal ANGPT2 fragments. We were unable to find a single protease that produces 3 fragments; however, CATK emerged as the only candidate protease that cleaved at 2 sites (Figure 2C) to produce predicted fragment sizes of 50.6 kDa (corresponding to amino acids 55S-496F in the protein) and 27.8 kDa (253E-496F). Activated macrophages upregulate CATK expression and release CATK protein through noncanonical secretion (34, 35). Figure 2G showed that recombinant CATK was sufficient to cleave recombinant human ANGPT2 into approximately 70 kDa, 50 kDa, and 25 kDa fragments, recapitulating the results of incubating CM-Mq^LPS^ with recombinant full-length ANGPT2 (Figure 1H). HEK293 cells transfected with expression vectors for ANGPT2 (55S–496F) and ANGPT2 (253E–496F) produced approximately 70 kDa band and 25 kDa bands, respectively, replicating the electrophoretic mobility of 2 major ANGPT2 cleavage products (Figure 2D).

To test if CM-Mq^LPS^ can cleave ANGPT2 at the predicted sites of 55S and 253E, we next performed mass spectrometry analyses of this reaction. The peptide SSSSPYVSNAVQR, spanning amino acids 55S to 67R, was detected in a mixture of ANGPT2 and CM-Mq^LPS^ while any peptide starting with 55S was absent from a reaction mixture of ANGPT2 and CM-Mq^Veh^ (Supplemental Tables 3 and 4). The peptide ETVNNLLTM, spanning amino acids 253E to 261M, was recovered in a mixture of ANGPT2 and CM-Mq^LPS^, while any peptide starting with 253E was absent from a reaction mixture of ANGPT2 and CM-Mq^Veh^ (Supplemental Tables 5 and 6). These results confirmed that peptides corresponding to predicted CATK cleavage sites arose specifically from a reaction of ANGPT2 with CM-Mq^LPS^ but not CM-Mq^Veh^.

To evaluate the cleavage reactions further, we designed 2 short ANGPT2 peptides within the predicted regions of CATK-induced cleavages: 42S–71L and 245Q–260T. These ANGPT2 peptides were incubated with recombinant CATK, and the cleavage patterns were analyzed by mass spectrometry (Figure 2, E and F). The polypeptide 42S–71L was cleaved at 6 sites (44T, 46L, 49E, 53C, 54R, and 66Q), including the predicted cleavage site of 55S. The polypeptide 245Q–260T was cleaved only at the predicted site of 253E. These results suggested that CATK may cleave ANGPT2 at multiple sites around 55S and a single site at 253E.

We next determined the role of ANGPT2 glycosylation. In the absence of deglycosylation, ANGPT2 proteins incubated with CATK generated bands of approximately 70, 50, and 25 kDa (Figure 2G). The sizes of deglycosylated ANGPT2 proteins were close to the predicted size based solely on amino acid sequence: full-length ANGPT2 was 54.8 kDa; ANGPT2 55S–496F was 50.6 kDa; and ANGPT2 253E–496F was 27.8 kDa. Only full-length ANGPT2 and ANGPT2 55S–496F were transformed into multiple bands when incubated with CATK; ANGPT2 253E–496F did not shift in size and was not converted to multiple bands. Deglycosylation did not abrogate the sensitivity of full-length ANGPT2 or ANGPT2 55S–496F to cleavage by CATK. Rather, deglycosylation enhanced the mobility of all ANGPT2 bands.

Together, these results supported a model of macrophage-released CATK cleaving ANGPT2 with multiple intermediate cleavage sites around 55S and a single final cleavage site at 253E. The calculated size of one major intermediate product was 50 kDa without glycosylation, and the size of the final dominant product was 25 kDa without glycosylation. Therefore, we named ANGPT2 cleaved around 55S and cleaved at 253E as cANGPT2_50_ and cANGPT2_25_, respectively. Recombinant CATK was most efficient at cleaving ANGPT2 in low pH (Supplemental Figure 2A) but maintained activity at physiological pH during inflammation. We further tested whether CATK cleaves ANGPT2_443_, which is the protein product when exon 2 lacks the alternative splicing variant of *ANGPT2*. CATK cleaved deglycosylated ANGPT2_443_ (which had a molecular weight of 48.9 kDa), resulting in multiple bands around approximately 45 kDa (estimated size, 44.7 kDa if cleaved at 55S) and a single band of approximately 28 kDa (estimated size, 27.8 kDa, the same weight as cANGPT2_25_) (Supplemental Figure 2C).

### CATK is responsible for cleaving ANGPT2 into Tie2 antagonist fragments.

**Figure 3:**
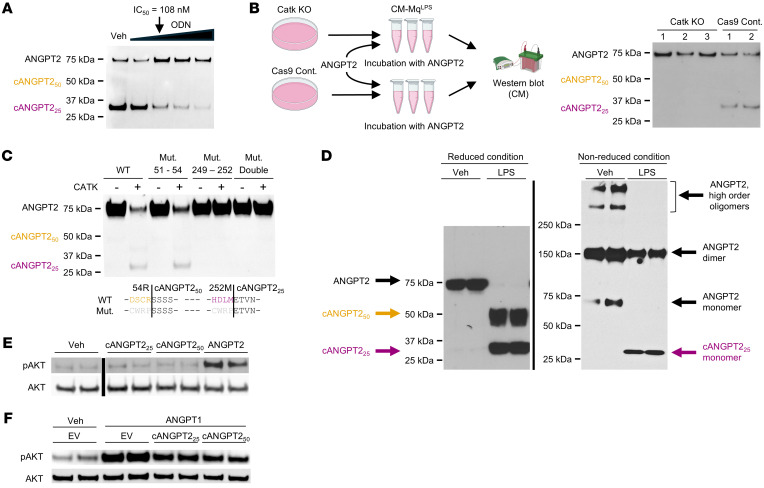
Cathepsin K is responsible for cleaving ANGPT2 into Tie2 antagonistic fragments. (**A**) RAW264.7 cells pretreated with vehicle or increasing concentrations of CATK inhibitor odanacatib (ODN, from left 12.5 nM, 125 nM, 1.25 μM, 12.5 μM). IC_50_ = 108 nM for mouse Catk (59). RAW264.7 cells were stimulated with LPS and then incubated with recombinant ANGPT2. ODN was added to the cells 1 hour prior to LPS and ANGPT2. (**B**) Incubation of recombinant ANGPT2 and CM-Mq^LPS^ harvested from CRISPR-mediated Catk-KO RAW264.7 clonal cell lines and isogenic Cas9 control cell lines. CRISPR-mediated targeting strategy and Catk-KO scores of the 3 cell lines are described in Supplemental Figure 4. (**C**) Incubation of recombinant CATK and conditioned media of HEK293 cells transfected with wild-type or mutant Angpt2 expression vectors at predicted Catk cleavage sites for cANGPT2_50_, for cANGPT2_25_, or for both sites (Double). (**D**) Left: Western analysis under reduced conditions (10% β-mercaptoethanol) of CM-Mq^LPS^ versus CM-Mq^Veh^ incubated with recombinant ANGPT2. Arrows indicate cANGPT2_50_ (orange) and cANGPT2_25_ (violet). Right: Same samples as shown to the left applied to SDS-PAGE followed by Western analysis under nonreducing conditions. Predicted oligomeric status is indicated. Following LPS treatment, the 150 kDa band may indicate heterotetramerization of 2 cANGPT2_50_ and 2 cANGPT2_25_ monomers. (**E**) Western analysis of phospho-AKT and total AKT in HUVECs stimulated with different recombinant versions of cleaved ANGPT2 protein (1,000 ng/mL). All samples were loaded on the same gel but were noncontiguous. (**F**) Western analysis of phospho-AKT and total AKT in HUVECs stimulated with ANGPT1 (80 ng/mL) and CM of control empty vector (EV) or cANGPT2-expressing HEK293 cells. Western analysis images are representative of at least 3 independent experiments.

To confirm if CATK is responsible for ANGPT2 cleavage, we tested the effect of odanacatib (ODN), a commercial selective inhibitor of CATK developed to treat bone resorption disorders. ODN exhibits nanomolar affinity for CATK, a 300-fold selectivity for CATK over cathepsin S, and >1,000-fold selectivity against other cathepsins (36). We observed that pretreatment of RAW264.7 cells with ODN dose-dependently reduced cleavage of ANGPT2 after LPS stimulation (Figure 3A). ODN had no effect on the appearance of cleaved Tie1 or Tie2 in the CM when HUVECs were inflamed (Supplemental Figure 3). Next, we applied CRISPR to create Catk-KO RAW264.7 clonal lines (Supplemental Figure 4, A and B). The CM-Mq^LPS^ from 3 separate Catk-KO RAW264.7 clonal cell lines did not cleave ANGPT2, while CM-Mq^LPS^ from isogenic Cas9-control RAW264.7 cells retained inducible ANGPT2 cleaving activity (Figure 3B). This result demonstrates genetically that CATK is required for the ANGPT2-cleaving activity of CM-Mq^LPS^.

Next, we mutated the cognate cleavage sites on ANGPT2 (Supplemental Figure 4C). When media from HEK293 cells transfected with vectors to express wild-type or mutated ANGPT2s was incubated with recombinant CATK, the results indicated that mutation around the 253E site was sufficient to prevent CATK cleavage (Figure 3C). Given (a) that multiple intermediate-sized bands between 50 and 70 kDa arose along with progressive intensification of a dominant 25 kDa sized band when ANGPT2 was reacted with CM-Mq^LPS^ (Figure 1G), (b) that earlier mapping experiments indicated several neighboring CATK sites in ANGPT2 between amino acids 43 and 71 (Figure 2E), (c) that the same mutation was introduced at both putative CATK sites (Supplemental Figure 4C), and (d) that the 50 kDa product appears as several bands (Supplemental Figure 2B), this result suggests that the 55S cleavage occurs at several nearby sites to generate intermediate fragments, whereas the 253E cleavage occurs at a single site to produce a C-terminal 25 kDa ANGPT2 fragment.

ANGPT2 is composed of 4 functional domains (Figure 2H). The FL domain is required for binding of ANGPT2 to the Tie2 receptor (17, 30). The CC domain enables ANGPT2 dimerization, and SCD promotes higher-order multimers of ANGPT2. The oligomerization state of ANGPT1 and ANGPT2 is considered important for their ability to cluster Tie2 and to induce cross-phosphorylation of Tie2. Optimal positioning of ANGPT receptor binding domains is crucial for Tie2 activation and determined by the oligomerization status of ANGPTs (33). To examine oligomerization of ANGPT2 fragments further, we performed Western analysis in reducing and nonreducing conditions for ANGPT2 after recombinant ANGPT2 was incubated with CM-Mq.

As expected under nonreducing conditions, full-length ANGPT2 formed dimers and, to a lesser extent, higher-order multimers (Figure 3D). For cANGPT2_25_ — which lacks both the SCD and CC domain — a monomer was observed; for cANGPT2_50_, the only other band in the nonreducing condition appeared at approximately 150 kDa, suggesting a heterotetramer composed of 2 cANGPT2_50_ and 2 cANGPT2_25_ or, less likely, a homotrimer of cANGPT2_50_ (Figure 3D). A pull-down assay with Tie2-Fc showed that both cANGPT2_50_ and cANGPT2_25_ bound the ectodomain of Tie2 (Supplemental Figure 5), consistent with both fragments containing the receptor binding FL domain.

Neither recombinant cANGPT2_50_ nor cANGPT2_25_ induced phosphorylation of AKT, one of the major signal transduction kinases downstream of Tie2 (37, 38), while full-length ANGPT2 induced AKT signaling in HUVECs (Figure 3E). Moreover, pretreatment with cANGPT2_50_ or cANGPT2_25_ inhibited ANGPT1-induced phosphorylation of Tie2 in HUVECs (Figure 3F). Taken together, full-length ANGPT2 formed high-order oligomers and stimulated Tie2, while cANGPT2_50_ and cANGPT2_25_ were not able to form high-order oligomers and antagonized Tie2. We therefore hypothesized that cleavage of ANGPT2 could be a proteolytic switch for converting its function from Tie2 agonist to antagonist in the context of sepsis. Characteristics of ANGPT2, cANGPT2_50_, and cANGPT2_25_ are summarized in Table 1.

### ODN inhibited ANGPT2 cleavage and CATK activity, and conferred beneficial organ effects in endotoxemic mice.

**Figure 4:**
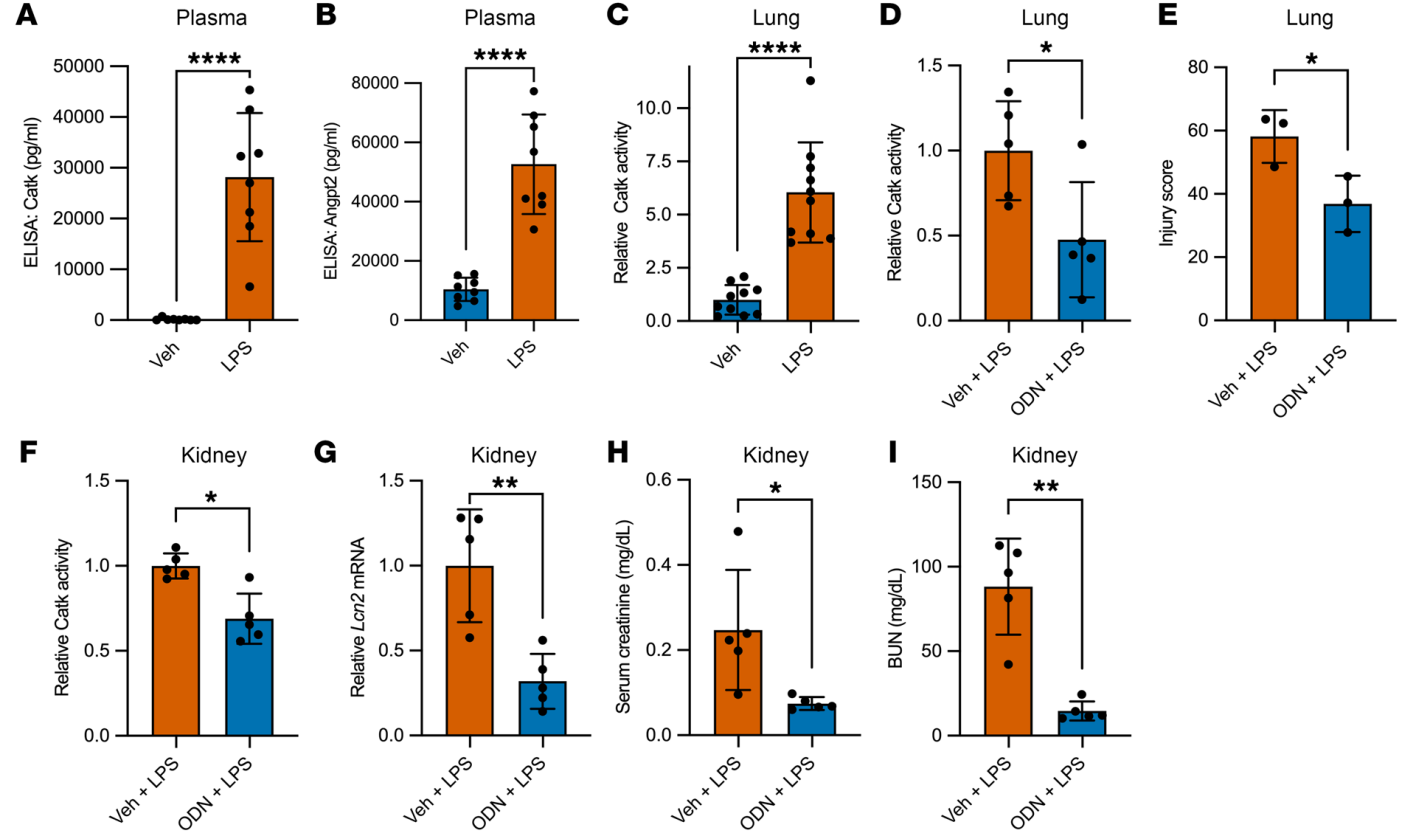
Inhibition of cathepsin K confers beneficial organ effects in endotoxemic mice. (**A** and **B**) Circulating Catk (**A**) and Angpt2 (**B**) protein levels 24 hours after LPS (10 mg/kg) administration (*n* = 8 mice per group). (**C**) Catk activity in the lungs of mice harvested 24 hours after LPS (10 mg/kg) administration (*n* = 10 mice per group). (**D**) Effect of ODN (20 mg/kg, 1 hour prior to LPS administration) on lung Catk activity 24 hours after LPS (10 mg/kg) administration (*n* = 5 mice per group). (**E**) Blinded scoring of histological lung injury as described in Methods. Representative images of H&E-stained lung sections are shown in Supplemental Figure 6, H and I. (**F**) Effect of ODN (20 mg/kg) on kidney Catk activity 24 hours after LPS (10 mg/kg) administration (*n* = 5 mice per group). (**G–I**) Markers of kidney injury: (**G**) whole-kidney *Lcn2* mRNA; (**H**) serum creatinine; and (**I**) blood urea nitrogen (BUN). *P < 0.05, **P < 0.01, ****P < 0.0001 by Mann-Whitney.

Circulating Catk (Figure 4A) and Angpt2 proteins (Figure 4B) were highly upregulated in the plasma of endotoxemic mice. This upregulation likely reflects release of Catk protein rather than transcriptional upregulation in macrophages or in vivo (Supplemental Figure 6, A–F). The approximate ratio of Angpt2 (52.7 ± 16.8 ng/mL, mean ± SD) to Catk (28.2 ± 12.6 ng/mL, mean ± SD) in the circulation was 1 to 0.5. In a reaction of recombinant proteins of ANGPT2 and CATK, we observed ANGPT2 cleavage at ANGPT2/CATK ratios of 1:0.1 to 1:1 (Supplemental Figure 6G). Enzymatic activity of Catk was evident in lungs and kidneys, 2 major target organs injured during sepsis; this activity was inhibited by ODN, which also improved lung and kidney injury (Figure 4, C–I, and Supplemental Figure 6, H and I). To test whether ANGPT2 cleavage was also inhibited in mice by ODN, we conducted gene transfer by hydrodynamic tail vein injection to express C-terminally His-tagged full-length ANGPT2 or recombinant versions of cANGPT2_50_ and cANGPT2_25_, followed by LPS administration and Western analysis of lung lysates. We probed with an anti-His antibody in order to minimize biased detection among the 3 peptides or nonspecific bands with the use of other immunoblotting antibodies (Supplemental Figure 6J). We observed robust appearance of cleaved ANGPTs, most notably cANGPT2_25_ (Supplemental Figure 6, K–M). Finally, to ask whether the in vivo cleaving activity of activated macrophages was specific to LPS or more generalized, we administered intraperitoneal thioglycolate — a canonical stimulus to generate inflammatory macrophages — and determined that peritoneal fluid from this model was sufficient to cleave recombinant ANGPT2 ex situ (Supplemental Figure 6N). Together, these results show that Catk is released during murine systemic inflammation, where it is sufficiently abundant to quantitatively cleave ANGPT2 in vivo.

### ODN improved survival in 2 sepsis mouse models in a cANGPT2_25_-dependent fashion.

**Figure 5:**
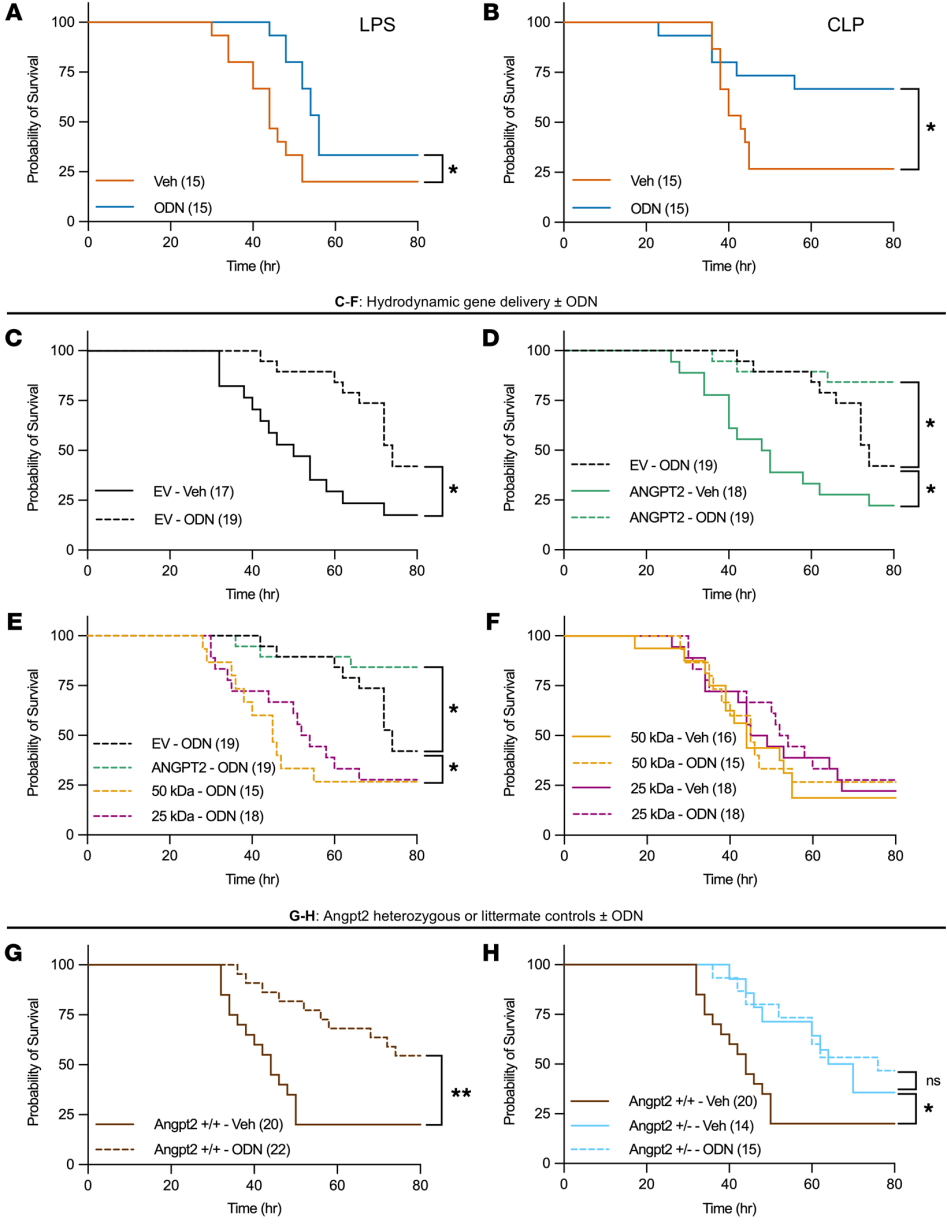
Cathepsin K inhibition improves survival in distinct sepsis models and is dependent on ANGPT2 expression and conversion to cleaved products. (**A** and **B**) Survival curves for mice after (**A**) LPS administration (10 mg/kg) or (**B**) cecal ligation puncture (CLP). Vehicle or ODN (20 mg/kg) was injected i.p. 1 hour prior to LPS administration or CLP surgery. (**C**–**F**) Survival curves after hydrodynamic gene transfer of indicated plasmids (10 μg DNA in 10% saline based on body weight, 4 hours prior to vehicle or ODN). Vehicle or ODN (20 mg/kg, i.p.) was administered 1 hour prior to LPS (10 mg/kg) injection. LPS injection time was recorded as time 0. Empty vector (EV) + ODN was used as the control group. (**G** and **H**) Survival curves for Angpt2 heterozygous or littermate wild-type control mice after ODN and LPS injection performed, as per **A**. The Angpt2+/+ + Veh group was used as the control group. Numbers in parentheses indicate mice per group. *P < 0.05, **P < 0.01 by log-rank test.

Next, we found that ODN improved survival in two distinct sepsis models: endotoxemia with LPS (Figure 5A) and the cecal ligation puncture (CLP) (Figure 5B). To test how full-length ANGPT2 and fragments modulate this effect, a series of gene transfer experiments was undertaken in conjunction with endotoxemia. First, the beneficial effect of ODN treatment was verified in these experiments (Figure 5C). Second, whereas full-length ANGPT2 enhanced the beneficial effect of ODN — consistent with an agonist function when uncleaved — full-length ANGPT2 was detrimental to survival in the absence of Catk inhibition, consistent with a Catk-dependent conversion of ANGPT2 in endotoxemia from agonist to antagonist (Figure 5D). Third, compared with the additional protective effect of full-length ANGPT2 in the presence of ODN, the recombinant cleaved ANGPT2 fragments were detrimental to survival despite ODN, consistent with a dominant antagonistic action (Figure 5E). Finally, the presence or absence of ODN did not change endotoxemia outcomes when either ANGPT2 fragment was overexpressed (Figure 5F). We also tested the effect of ODN in Angpt2 heterozygous mice. In the absence of ODN, Angpt2 genetic depletion had the expected beneficial effect on survival (25). In the presence of ODN, a significant survival benefit was evident in endotoxemic wild-type littermates, but not in Angpt2 heterozygous mice (Figure 5, G and H). These data therefore propose, through complementary loss- and gain-of-function genetic experiments, that Catk inhibition during sepsis enhances survival and that ODN’s protective effect depends on its ability to prevent the generation of detrimental ANGPT2 fragments.

### Detection of cleaved ANGPT2 in patients with sepsis.

**Figure 6:**
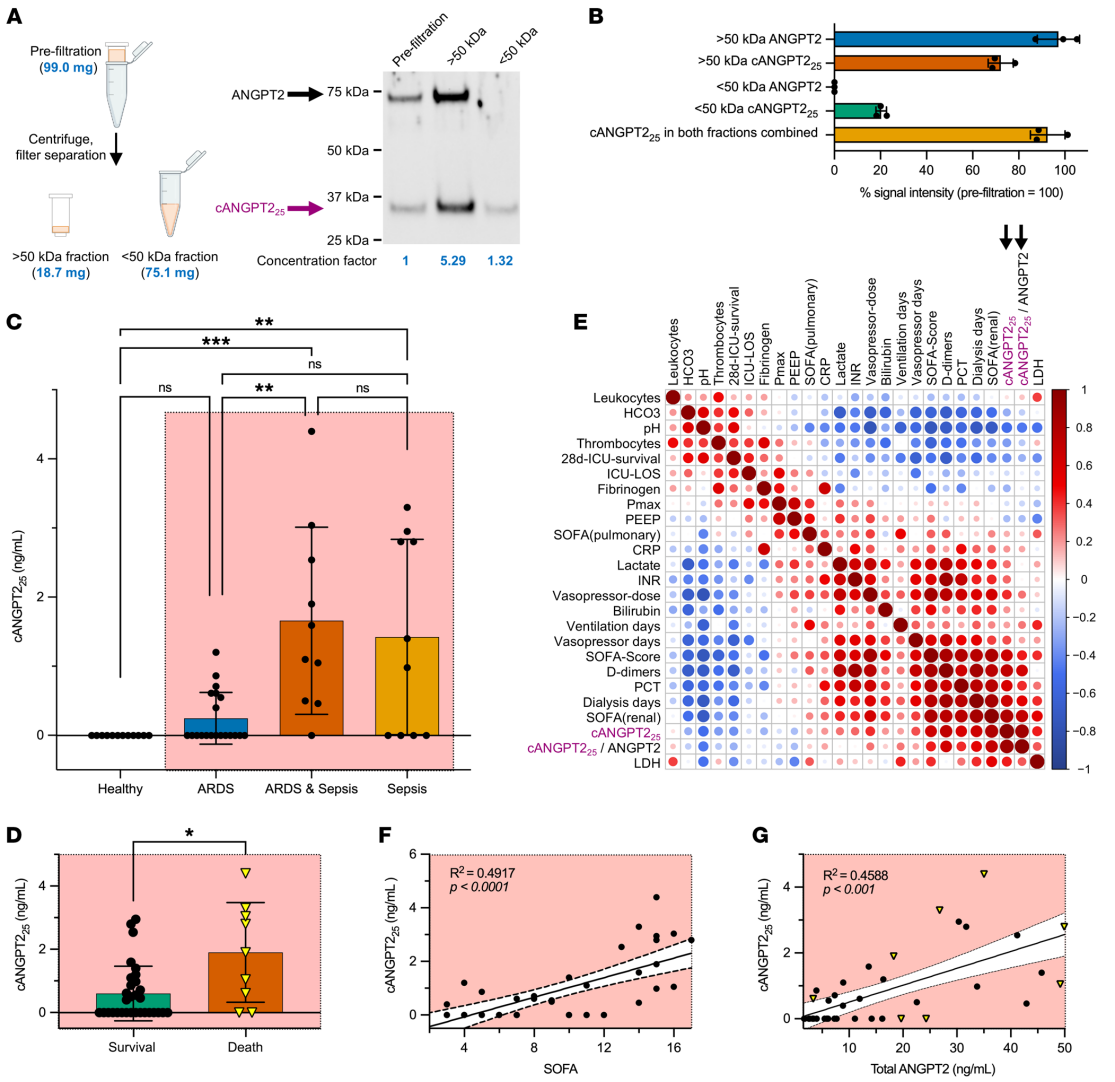
Circulating cleaved ANGPT2 is associated with adverse outcomes in critically ill humans. (**A** and **B**) Western analysis of molecular weight column filtration (cut off 50 kDa) to separate full-length ANGPT2 and cANGPT2_25_. (**A**) His-tagged recombinant ANGPT2 was incubated with CM-Mq^LPS^ and separated into a >50 kDa and a <50 kDa fraction. ANGPT2 and cANGPT2_25_ were visualized with anti-His antibody. Fraction concentration factor was calculated by determining the weight of retentate (18.7 mg) and filtrate (75.1 mg) versus prefilter weight (99 mg). Representative blot of 3 independent experiments depicted, along with (**B**) quantified band signal intensities corrected by concentration factors and normalized to prefiltration band intensity set to 100% (mean ± SD, *n* = 3): >50 kDa ANGPT2, 97.27% ± 9.17%; >50 kDa cANGPT2_25_, 72.19% ± 5.40%; <50 kDa ANGPT2, 0%; <50 kDa cANGPT2_25,_ 20.37% ± 2.30%; and cANGPT2_25_ detected in both fractions combined, 92.56% ± 7.49%). (**C**) cANGPT2_25_ (ng/mL) in sera of healthy individuals acting as controls (healthy, *n* = 10), patients in the ICU with a primary diagnosis of acute respiratory distress syndrome (ARDS, *n* = 20), ICU patients with a primary diagnosis of ARDS and sepsis (ARDS & Sepsis, *n* = 10), and ICU patients with a primary diagnosis of sepsis (Sepsis, *n* = 10) (Kruskal-Wallis, **P < 0.01, ***P < 0.001). (**D**) cANGPT2_25_ (ng/mL) stratified by survival (*n* = 31) versus death (*n* = 9) 28 days after diagnosis of ARDS, ARDS and sepsis, or sepsis (Mann Whitney, *P < 0.01). (**E**) Spearman’s correlation plot showing ICU patients with diagnoses of ARDS, ARDS and sepsis, or sepsis (*n* = 40) comparing major parameters from blood tests and other assessments, as defined against absolute concentration of cANGPT2_25_ (ng/mL) and the ratio of cANGPT2_25_ to the total amount of ANGPT2. Color indicates strength of association by correlation coefficient. ICU LOS, ICU length of stay; Pmax, peak inspiratory pressure; PEEP, peak end-expiratory pressure; SOFA, sequential organ failure assessment score; INR, international normalized ratio measuring blood coagulation; PCT, procalcitonin; LDH, lactate dehydrogenase. (**F** and **G**) Correlation between cANGPT2_25_ (ng/mL) in ICU patients and SOFA score (**F**) and total ANGPT2 (ng/mL). Data in **G** are from **E**. Triangles indicate death within 28 days of diagnosis.

Finally, we sought to test whether ANGPT2 cleavage arises in critically ill humans and, if so, whether cleavage is associated with adverse clinical outcomes. First, we developed a size-filtering protocol suitable for biosamples for the detection of ANGPT2 fragments. We confirmed the ability to recover cANGPT2_25_ and exclude full-length ANGPT2 (Figure 6, A and B). Although the recovery rate of cANGPT2_25_ was approximately 20%, full-length ANGPT2 was completely excluded in the <50 kDa fraction that was applied to ELISA. Using serial dilutions of cANGPT2_25_ in a widely used commercial ELISA, we confirmed the ELISA’s ability to detect the C-terminal fragment of ANGPT2 and its linear performance in the target range (Supplemental Figure 7, A and B). Then, we evaluated sera collected from healthy volunteers and from patients within 24 hours of admission to the ICU for sepsis, acute respiratory distress syndrome (ARDS), or the combination of both (Table 2). Compared with healthy individuals acting as controls, patients with sepsis or the combination of ARDS and sepsis exhibited elevated circulating cANGPT2_25_ (Figure 6C). Within the ICU group, 28-day survival was associated with lower levels of cANGPT2_25_ compared with those who died of sepsis during this period (Figure 6D). An extensive analysis of associations was undertaken in the ICU cohort samples to evaluate the relationships among clinical parameters, laboratory measurements, the ratio of circulating cANGPT2_25_ to total ANGPT2 (Supplemental Figure 7C), and the concentration of cANGPT_25_ (Figure 6E). A hotspot for positive correlations was noted in the lower right quadrant of the heatmap, which depicts multiple significant associations between cANGPT_25_ and a spectrum of clinical and molecular indicators of severe illness, including the sequential organ function assessment (SOFA) score, a commonly used score of organ failure severity (Figure 6F); D-dimers, a standard measurement of consumptive coagulopathy (Figure 6E); and procalcitonin, a molecular biomarker of bacterial infection; duration of dialysis, implemented for severe acute kidney injury; a SOFA sub score for renal injury (Supplemental Figure 7D); and cANGPT2_25_. Notably, the heatmap in Figure 6E also confirmed well-accepted relationships: e.g., a strong negative association between arterial pH and the duration of dialysis (lower left or top right) and positive association between INR and D-dimers (middle right or lower center), elevation in either of which would support a diagnosis of consumptive coagulopathy in the ICU. The cANGPT2_25_ concentration displayed strong positive relationships with vasopressor dose and the hemodynamic SOFA sub score (Supplemental Figure 7, E and F). As expected, the total ANGPT2 concentration was correlated to the concentration of cANGPT2_25_ (Figure 6G). The small size of the endogenous ANGPT2 fragment was also suggested by its appearance in the urine of a separate cohort of pediatric patients with sepsis (Supplemental Figure 8 and Supplemental Table 7) since the human kidney enables filtration of proteins smaller than 60 kDa. These results propose that ANGPT2 fragments are present in sepsis, associated with organ dysfunction, and linked to death during hospitalization.

## Discussion

ANGPT2 was originally identified as an endogenous Tie2 antagonist (15). Subsequent work proposed that ANGPT2 could exert an autocrine agonist effect (40). Attempts to resolve this paradox have gained urgency with markedly elevated ANGPT2 levels being reported in diverse infection- and inflammation-associated conditions, including sepsis and ARDS, anthrax, disseminated coagulopathy, falciparum malaria, and, most recently, severe COVID (7, 25, 39, 41–46). The present results describe a potentially new proteolytic mechanism arising from inflammation to transform ANGPT2 into a Tie2 antagonist and a new action of a clinical-stage compound to mitigate adverse outcomes of sepsis in a CATK- and ANGPT2-dependent fashion.

Landmark studies from the 1980s illuminated the central role of proteolysis in the pathogenesis of sepsis (47, 48). More recent observations have linked systemic proteolysis to adverse outcomes in sepsis and even implicated exocrine pancreatic enzymes (49, 50). However, to our knowledge, a direct pathogenic role for CATK in sepsis has not been described previously.

Selective proteolysis is separately appreciated to regulate vascular endothelium in health and disease. This is dramatically manifest in thrombotic thrombocytopenic purpura, a condition of genetic or acquired deficiency for the protease that cleaves the endothelium-derived platelet binder von Willebrand factor (51). Loss of this proteolytic activity can result in a profound systemic disorder of platelet clumping. Notably, both von Willebrand factor and ANGPT2 are made and stored in the same Weibel-Palade bodies of vascular endothelium prior to exocytosis (52).

CATK is among a group of cysteine proteases normally localized to lysosomes and tasked with disposal of intracellular proteins. Although cathepsins display highest activity in the lysosome’s acidic environment, CATK possesses measurable activity at neutral pH (34, 35). Proteomic studies have established that CATK and other cathepsins are released by activated immune cells both through conventional secretion as well as cell lysis arising from pyroptosis (53, 54). ODN was developed for osteoporosis to target the CATK activity of osteoclasts (55). While safety concerns with chronic usage discouraged its development despite efficacy in a large phase III trial, the present findings identify a rationale to explore ODN’s utility in acute settings.

Several reports have examined the rapid loss of Tie2 signaling in sepsis. In endotoxemia, Korhonen et al. demonstrated that Tie2 activation — dependent on either ANGPT1 or ANGPT2 — becomes attenuated from rapid cleavage of Tie1 (13). Analogously, we found that Tie2 undergoes proteolytic cleavage by matrix metalloprotease 14 in sepsis (56). Finally, Souma et al. showed that Tie2 signaling in the lymphatic vasculature relies on ANGPT2 and noted that this specialized vasculature lacks expression of a protein tyrosine phosphatase called VE-PTP that is responsible for inhibiting Tie2 signaling (18).

All of these may contribute to the striking phenotypic shift of vascular endothelium during sepsis. However, the distinct antagonistic function of ANGPT2 on vascular endothelial Tie2 during sepsis was demonstrated by Han et al. They found that ANGPT2 can be converted from a Tie2 antagonist during sepsis into an agonist by administration of a unique antibody that forces oligomerization of native ANGPT2 (12). The dependence of ligand action on oligomerization aligns with earlier structural and stoichiometric studies, including N-terminal domain swap experiments between ANGPT1 and ANGPT2, that implicate N-terminus–dependent oligomerization as the basis for the differential activity of ANGPT1 versus ANGPT2 at Tie2 (32, 33).

The present results offer a mechanism to elucidate how inflammation unveils ANGPT2’s deleterious actions. As shown previously, excess ANGPT2 was deleterious in experimental sepsis (11) but actually beneficial in sepsis when its cleavage was prevented. Furthermore, the benefit of CATK inhibition was overcome by excess cleaved ANGPT2. Conversely, genetic depletion of Angpt2 abrogated the benefit of ODN. While released CATK is likely to cleave many soluble proteins in sepsis, our data support that ANGPT2 cleavage is required for the full lethality of sepsis and sufficient to exacerbate septic lethality even when CATK cleavage is inhibited.

Limitations of the present work highlight opportunities for future studies. First, the secretome of activated immune cells contains many proteolytic enzymes (53), thus understanding which ones exert significant effects in the many distinct settings that involve activated immune cells requires careful dissection. Even ANGPT2 itself may be cleaved by other proteases released from activated immune cells. Conversely, CATK is likely to have more than one target in vivo: for example, CATK can also cleave ANGPT1 (Supplemental Figure 9). Second, our studies focused on LPS as a trigger for CATK secretion. How LPS’s secretagogic activity compares with that of other agonists of Toll-like receptor 4 or even unrelated macrophage-activating pathways merits further exploration. Activated macrophages may release other cathepsins — and because ODN could inhibit other cathepsins at the concentration applied here — the cellular and murine results need to be verified with targeted KO of Catk. Third, while we report and model 2 distinct fragments of 50 and 25 kDa, our results strongly suggest that the 50 kDa band may be several transiently appearing peptides, whereas the 25 kDa fragment appears to be a single cleavage product based on electrophoretic mobility, cleavage mapping by MS, and mutagenesis experiments. We found that ANGPT2 fragments continue to bind Tie2 yet lose the ability to oligomerize. The present approaches to mapping cleavage sites on ANGPT2 included mass spectrometry performed on candidate short peptides, a mass spectrometry survey of all products arising from a reaction of activated versus control CM with ANGPT2, and mutagenesis of the cognate sites on ANGPT2. An Edman degradation approach to sequence isolated fragments could provide definitive N-terminal amino acid identification. More broadly, elucidation of the protein chemistry of cleaved ANGPT2 fragments may also facilitate novel approaches to prevent fragment formation or fragment-dependent Tie2 antagonism. Recovery of endogenous cleaved ANGPT2 is proposed by our human ELISA results, but a reporter mouse could help quantify this phenomenon in multiple inflammatory models. Finally, when considered with the present results, previous reported benefits of the ANGPT2-clustering antibody in experimental sepsis (12) suggest that ANGPT2 may exist in both whole and cleaved forms during sepsis, with the former being amenable to antibody-driven therapeutic clustering or other depletion strategies and the latter requiring therapeutic elimination. Combination treatment with ANGPT2 clustering and CATK inhibition remains to be tested.

In summary, the present results apply genetic and pharmacologic approaches in loss- and gain-of-function studies to support a model of acquired Tie2 antagonism arising from inflammation-induced and non-cell-autonomous ANGPT2 cleavage by CATK released from activated macrophages. This pathway appears to be both measurable and targetable, thus opening avenues for the development of new ANGPT- and CATK-based biomarkers and therapeutics for sepsis and related inflammatory conditions.

## Methods

### Sex as a biological variable.

In mouse studies utilizing hydrodynamic tail vein gene transfer, we examined male FVB mice. This choice was made in order to limit experimental variation atop the manipulations of drug and plasmid injection. For Angpt2^+/–^ mouse experiments, we examined animals of both sexes. The ratio of male to female mice was statistically balanced. Study participants were selected without consideration of their sex.

### Materials.

LPS and PMA were purchased from Sigma-Aldrich. Recombinant TNF-α and mouse and human ANGPT2 were purchased from R&D Systems. CATK inhibitor ODN was purchased from Santa Cruz. Recombinant CATK was purchased from Enzo Life Sciences. The CATK activity assay kit was purchased from BioVision. The Amicon Ultra centrifugal filters (MWCO, 10 kDa and 50 kDa) were purchased from Millipore. Brewer thioglycolate medium was purchased from Millipore. Details regarding antibodies are provided in Supplemental Table 8.

### Mouse studies.

Male 8- to 10-week-old FVB mice were purchased from The Jackson Laboratory. For Angpt2^+/–^ mouse experiments, the background strain was C57BL/6J, and littermate wild-type controls were studied. Mice were acclimated to the animal facilities for at least 1 week before experiments. For survival experiments, mice were administered LPS *E*. *coli* serotype O111:B4 intraperitoneally (10 mg/kg) or underwent CLP performed as previously described (57). To induce overexpression of ANGPT2 isoforms, 10 μg mammalian vector DNA dissolved in sterile saline (10% body weight) was injected via tail vein over 8–10 seconds using a 30.5-gauge needle under isoflurane anesthesia. ODN stock solution was dissolved in DMSO at 100 mg/mL. Diluted ODN in corn oil (1:20) was administered at 20 mg/kg 1 hour prior to LPS administration or CLP surgery. Animals were examined every 2 hours after intervention and assigned a distress score using an IACUC-approved scale. Once mice crossed the approved threshold, they were humanely euthanized, and death was considered as an endpoint for survival analysis.

### Mouse lung histology analysis.

Mouse lungs were harvested from anesthetized mice and immersion fixed in 20 volumes of 10% neutral buffered formalin. Following 48 hours of constant agitation, lungs were briefly rinsed and then dehydrated, cleared, and paraffin embedded in standard fashion. Serial 5 μm sections were stained with H&E. A minimum of 5 fields for each lung slide was examined and scored for pathological injury according to the guidelines of the American Thoracic Society (58).

### Cells and conditioned CM.

HUVECs (Lonza) were cultured according to the manufacturer’s instructions with 3–6 passages utilized for the experiments. RAW264.7 murine macrophage-like cells and HEK293 cells (ATCC) were cultured in DMEM (Gibco) supplemented with 10% FBS (ATLANTA Biologicals). To produce CM of LPS or vehicle-stimulated RAW264.7 cells (CM-Mq^LPS^ and CM-Mq^Veh^), confluent RAW264.7 cells were stimulated with LPS (100 ng/mL) in serum-free DMEM. One hour after stimulation, media was exchanged with serum-free DMEM after washing cells twice with warm PBS. CM was harvested 24 hours after LPS stimulation and centrifuged at 250*g* for 5 minutes to remove dead cells. Supernatant was stored at –80°C until use. 90% confluent HUVECs were stimulated with 10 ng/mL PMA in starvation media comprising EBM-2 with 0.25% FBS. 24 hours after PMA activation, HUVECs were stimulated with LPS (1 μg/mL) or vehicle for 4 hours. Then, recombinant ANGPT2 (500 ng) was added.

### Preparation of mouse tissues.

Animals were euthanized by exsanguination under anesthesia, followed by rapid collection of organs into liquid nitrogen for further analysis. 20–30 mg of mouse lung was homogenized in ice-cold nondenaturing lysis buffer (20 mM Tris-HCl pH 8.0, 137 mM NaCl, 1% Triton X-100, 2 mM EDTA) supplemented with protease inhibitor cocktail (Complete Mini, EDTA-free, Sigma-Aldrich) and phosphatase inhibitor (PhosSTOP EASYpack, Sigma-Aldrich). Lysates were sonicated and centrifuged at 8,000*g* for 15 minutes at 4°C, and supernatant was collected.

Additional details regarding experimental methods are provided in Supplemental Methods.

### Illustrations.

Illustrations were made by using BioRender.

### Statistics.

Statistical significance was evaluated using the Mann-Whitney *U* test or 1-way ANOVA followed by multiple comparisons, unless otherwise specified. Survival data were analyzed by log-rank test and visualized by Kaplan-Meier curves. The correlation plot was done with R Statistical Environment V4.3.1 program. All experimental results are presented as the mean ± SD, and a 2-tailed *P* value of less than 0.05 was considered to indicate statistical significance. GraphPad Prism 9.55 was used to depict the graphs and calculate the statistics.

### Study approval.

All animal experiments were approved by the IACUC of UT Southwestern Medical Center. Human samples were obtained under protocols approved by the Institutional Review Board at UT Southwestern Medical Center and Department of Respiratory Medicine and German Centre of Lung Research, Hannover Medical School. Written informed consent was obtained from participants.

### Data availability.

Values for all data points in figures are reported in the Supporting Data Values file. Additional information and detailed methods are available upon request to the corresponding author.

## Author contributions

TS designed, conducted, and analyzed experiments. SMP designed and supervised the study. EL, SC, TOI, SL, GB, VE, AJC, and VV conducted wet-bench experiments. VE, BS, SD, AJC, MCS, and BMF designed, conducted, and analyzed human studies. TS and SMP wrote the manuscript with input from all authors.

## Supplementary Material

Supplemental data

Unedited blot and gel images

Supporting data values

## Figures and Tables

**Table 1:**
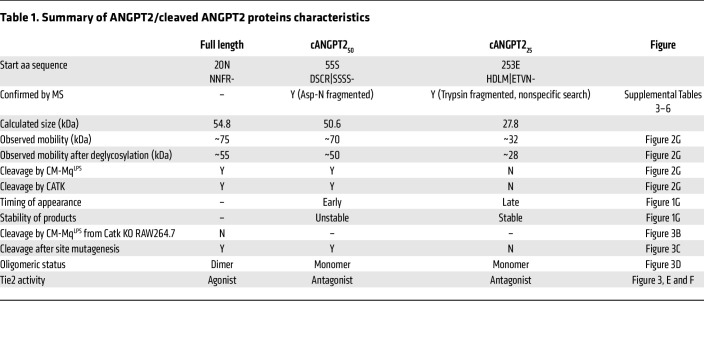
Summary of ANGPT2/cleaved ANGPT2 proteins characteristics

**Table 2:**
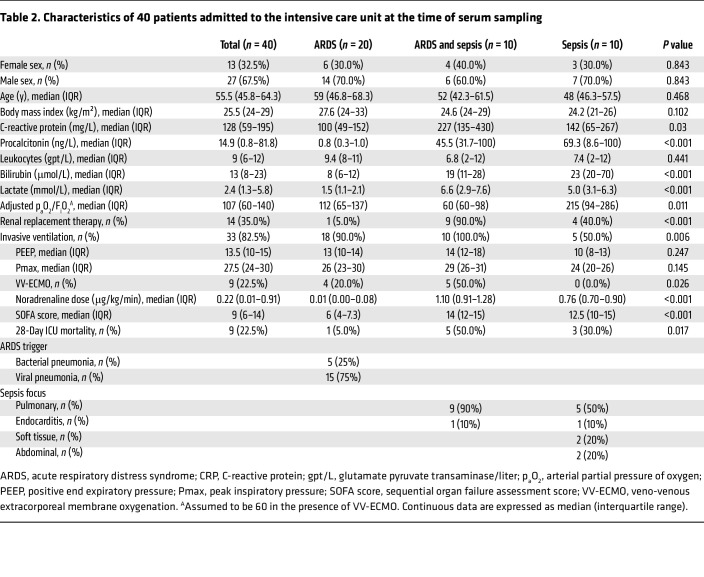
Characteristics of 40 patients admitted to the intensive care unit at the time of serum sampling

## Version 1 03/03/2025

In-Press Preview

## Version 2 04/15/2025

Electronic publication
