# Supplementary material for: Cathepsin K cleavage of angiopoietin-2 creates detrimental Tie2 antagonist fragments in sepsis
Source: J Clin Invest. 2025 Mar 3;135(8):e174135. doi: 10.1172/JCI174135 (PMC11996858; doi:10.1172/JCI174135)
Supplement: Supplemental data [file jci-135-174135-s309.pdf]

Supplemental Materials for

Cathepsin K cleavage of Angiopoietin-2 creates detrimental Tie2 antagonist fragments in sepsis

Authors: Takashi Suzuki[1], Erik Loyde[1], Sara Chen[2], Valerie Etzrodt[1], Temitayo O. Idowu[1], Amanda J. Clark[1,3], Marie Christelle Saade[1], Brenda Mendoza Flores[1], Shulin Lu[2], Gabriel Birrane[4], Vamsidhara Vemireddy[1], Benjamin Seeliger[5,6], Sascha David[7], Samir M. Parikh[1,2,8]

[1]Division of Nephrology, Department of Medicine, University of Texas Southwestern, Dallas, Texas, USA.
[2]Center for Vascular Biology Research, Beth Israel Deaconess Medical Center and Harvard Medical School, Boston, Massachusetts, USA.
[3]Division of Pediatric Nephrology, Department of Pediatrics, University of Texas Southwestern and Children's Medical Center, Dallas, Texas, USA.
[4]Division of Experimental Medicine, Department of Medicine, Beth Israel Deaconess Medical Center and Harvard Medical School, Boston, Massachusetts, USA.
[5]Department of Respiratory Medicine, Hannover Medical School, Germany
[6]Biomedical Research in End-Stage and Obstructive Lung Disease (BREATH), Hannover Medical School (MHH), German Center for Lung Research (DZL), Hannover, Germany
[7]Institute of Intensive Care Medicine, University Hospital Zurich, Zurich, Switzerland
[8]Department of Pharmacology, University of Texas Southwestern, Dallas, Texas, USA.

To whom correspondence should be addressed:
Samir M. Parikh
University of Texas Southwestern
5323 Harry Hines Blvd
Dallas, TX 75390
Email: samir.parikh@utsouthwestern.edu

Supplemental Methods

CRISPR. SgRNAs were designed using Synthego design tool and a 3-guide strategy (AAAUCUCUCGGCGUUUAAUU, GGACACCAAGAGAGGCCUCC, UGGGCAGGCCUGGAUCUGAG). SgRNAs were incubated with recombinant spCas9 at room temperature for 10 minutes before nucleofection into 1 x 10^6 RAW264.7 cells using the Amaxa nucleofector with the Lonza nucleofector Kit V. Cells were cultured after nucleofection in standard DMEM with 10% FBS. After 24 hours, DNA was isolated from cells using the DNAEasy Kit (Qiagen) and a 450bp region over the mutated segment was amplified via PCR (FWD CCTTTGCATAAGATTTGTGTATGTACTC, REV GACCCTGCCTTCCCACTCT). The PCR product was purified using Qiagen PCR purification kit and sequenced (Seq primer TTTCCCAGTTAGATTGTGTTTTCTGTTTAT) using the Sanger sequencing core at UT Southwestern. The resulting sequences were analyzed using Synthego's ICE analysis tool (https://ice.synthego.com/#/). Isogenic clones were made by splitting the cells to single cell populations and allowing proliferation into clonal colonies before DNA isolation and sequencing as above.

Vector construction. Primer sequences (**Supplemental Table 1**) with appropriate restriction enzyme sites were used for construction of ANGPT2 expression vectors. Q5 High-Fidelity DNA polymerase (New England BioLabs) was used to amplify ANGPT2 cDNAs. HUVECS were used to source full length ANGPT2 cDNA. PCR cycle conditions to clone full length ANGPT2 were: 98°C for 10 seconds, 68°C for 20 seconds, and 72°C for 50 seconds for 30 cycles. After ANGPT2 sequence was confirmed, PCR products were digested with restriction enzymes of KpnI and EcoRI followed by ligation into pcDNA3.1 vector (Life Technologies). Using the completed full length ANGPT2 expression vector as template DNA, overhang extension PCR technique was utilized to construct cleaved ANGPT2 expression vectors by inserting a start codon (ATG) and IL-2 signal peptide coding sequence (atgtacaggatgcaactcctgtcttgcattgcactaagtcttgcacttgtcacaaacagt) in front of cleaved ANGPT2 coding sequences. For the purification of proteins, 6x His tag coding sequence was added to the 3'-end.

Expression and purification of recombinant ANGPT2 proteins. HEK293 cells were seeded to 6 well plates (3 x 10^5 cells / well). When the cells reached 70-80% confluency, culture media was changed to serum-free DMEM before transfection. Using FuGENE 6 (Promega) ANGPT2 and $ANGPT2_{443}$ expression vectors were transfected to the cells according to the manufacturer's instructions. CM was collected 48 hours post-transfection and centrifuged at 1,200 rpm for 5 minutes to remove dead cells. Supernatant was stored at -80°C until use. Recombinant ANGPT2, $cANGPT2_{50,}$ and $cANGPT2_{25}$ proteins were prepared using the Expi293 Expression system (Invitrogen). 150 µg of expression vector and 480 µl of ExpiFectamin 293 Reagent were mixed in 17.4 mL of Opti-MEM (Invitrogen). The mixture was incubated at room temperature for 15 minutes then added to 3 x 10^6 Expi293 suspension cells in 150 mL of Expi293 Expression Medium. 24 hours post-transfection, 0.9 mL of ExpiFectamin 293 Transfection Enhancer 1 and 9 mL Enhancer 2 were added to the cell culture flask. Cells were cultured in a shaker incubator at 37°C with a humidified atmosphere of 8% CO_2 in air. Five days post-transfection, harvested culture supernatants were centrifuged to remove cells and debris then stored at -80°C until the next purification step. ANGPT2 proteins were purified by immobilized metal affinity chromatography (IMAC) on a nickel-nitrilotriacetic acid (HisPur™ Ni-NTA Superflow Agarose, Thermo) column. The concentration of purified proteins was measured using BCA assay kit (Pierce).

Tie2-Fc pulldown assay. 500 µL of $CM\text{-}Mq^{LPS}$ was incubated with 500 ng of recombinant ANGPT2 protein at 37°C for 20 hours followed by concentration using an Amicon Ultra (10 kDa) centrifugal filter. The concentrated reaction mixture was incubated with 500 ng of recombinant human Tie2-Fc protein (R&D Systems) in 500 µL of PBS supplemented with 8 mM $CaCl_2$ and protease inhibitor cocktail (Sigma Aldrich) at 4°C overnight with gentle agitation. As a negative control, the concentrated reaction mixture was incubated with human IgG isotype control (Invitrogen). Following incubation with Tie2-Fc or IgG isotype control, the mixture was incubated with Dynabeads Protein G at room temperature for 10 min. Bead-conjugated complexes were washed with PBS-T (0.02% Tween 20) four times, and bound proteins were eluted with glycine elution buffer (100 mM Glycine, pH 2.2) and separated by SDS-PAGE.

Western blot. Collected conditioned media (CM) was centrifuged to remove debris, and supernatants were applied to SDS-PAGE gel. Cell lysates were prepared by homogenization in ice-cold lysis buffer (20 mM Tris-HCl, pH 8, 137 mM NaCl, 1% NP-40, 2 mM EDTA) supplemented with protease inhibitor (Sigma Aldrich) and phosphatase inhibitor (Sigma Aldrich). Lysates were sonicated and centrifuged at 8,000 x *g* for 15 minutes at 4°C, and supernatants were collected. Using BCA assay Kit (Pierce), protein concentrations in supernatants were measured and 10 μg of protein were separated by SDS-PAGE using NuPAGE Bis-Tris gels (4-12% gradients) with MES SDS running buffer or NuPAGE Tris-Acetate gels (3-8% gradients) with MOPS running buffer (ThermoFisher Scientific). The proteins were transferred to nitrocellulose membranes, blocked for 1 hour (SuperBlock T20, ThermoFisher Scientific), immunoblotted with specific primary antibodies, and incubated with the appropriate secondary antibody conjugated with horseradish peroxidase. Bands were visualized using ECL kit (ThermoScientific). To investigate oligomeric status of ANGPT2, western blot was performed under nonreducing conditions without reducing agents.

Deglycosylation of ANGPT2 proteins. Recombinant ANGPT2 proteins were deglycosylated using Protein Deglycosylation MIX II kit (NEB). Buffer 1 was used to minimize the ANGPT2 (full-length) doublet formation (**Supplemental Figure 10**). Buffer 2 was used for deglycosylation of $cANGPT2_{50}$ and $cANGPT2_{25}$ and no doublet formation was observed (**Figure 2G**).

RT-qPCR. Total RNA of HUVECs was extracted using RNeasy Mini kit (QIAGEN). Total RNA of mouse tissues was extracted using TRIzol (Invitrogen), followed by clean-up using the RNeasy Mini Kit with on-column DNase digestion (QIAGEN) per the manufacturer's instructions. Total RNA (1 μg) was then reverse transcribed to cDNA using SuperScript III Reverse Transcriptase (Invitrogen). Quantitative PCR reactions were performed using SYBR-green reaction mix (Qiagen) in duplicate using ABI QuantStudio 6 Flex System (Applied Biosystems) and the appropriate primers (**Supplemental Table 2**). Relative expression levels were determined using the comparative threshold method.

Serum markers of renal injury. Mouse BUN was measured using a commercially available assay (Invitrogen). Serum creatinine was measured using capillary electrophoresis in the UT Southwestern Renal Physiology Core.

Transendothelial Electrical Resistance assays. Transendothelial Electrical Resistance (TEER) was measured using an electric cell-substrate impedance sensing system (ECIS) (Applied Bio- Physics Inc). Values were pooled at discrete time points and plotted versus time. The normalized TEER was derived by dividing each condition's endpoint resistance by its starting resistance. A representative time point (20 hours) was chosen after stabilization of relative resistance due to addition of CM-Mq.

Reaction of recombinant ANGPT2 and recombinant CATK in test tube. 200 ng of recombinant ANGPT2 was incubated with 200 ng of recombinant CATK protein in 50 µL of pH 5.5 solution (50 mM Sodium Acetate pH 5.5, 50 mM Sodium Chloride, 0.5 mM EDTA, 5 mM DTT) at 37°C for 5 minutes. Immediately after the reaction, CATK was deactivated by heating at 95°C for 5 minutes. 16 µL of the reaction mixture was applied to SDS-PAGE gel for western blot of ANGPT2.

Measurement of Catk activity. The Catk activity in mouse lung was evaluated using Cathepsin K Activity Fluorometric Assay kit (BioVision). 20-30 mg of mouse lung was homogenized in the provided lysis buffer, and 200 µg of lung lysate was used for the assay.

Mass spectrometry. Two ANGPT2 polypeptides of 42S-71L and 245Q-260T were synthesized at Boston Molecules Inc. (Waltham, MA) and reconstituted in 80% water, 20% Acetonitrile, 0.1% Formic Acid. 1 µg of the polypeptide was incubated with 2 ng of recombinant CATK in 10 µL of pH 5.5 solution at 37°C for 5 minutes. 3 µL of 1M Tris-HCl pH 8.0 was added immediately after the reaction for neutralization to deactivate CATK activity. Cleavage patterns of ANGPT2 polypeptides were analyzed by LC/MS fragmentation. Reaction of ANGPT2 and CM-Mq was performed in Protein LoBind Tube (Eppendorf). 2 µg of Recombinant ANGPT2 was incubated with 1 mL of protease cocktail (cOmplete Mini EDTA-free, Roche) supplemented CM-Mq at 37°C for

24 hours. Speed-vacuum concentrated samples were fragmented by either Asp-N or trypsin for amino acids sequencing via mass spectrometry.

Validation of circulating Angpt2 and CatK in endotoxemia mice. Angpt2 and Catk protein level in serum was measured using Angpt2 ELISA kit (R&D, MANG20) and Catk ELISA kit (Novus Biologicals, NBP3-00426).

ELISA validation of ANGPT2 proteins. 100 µL of serum or 500 µL of urine was applied to Amicon Ultra (50 kDa), and the flow through was collected after centrifugation. Concentration of $cANGPT2_{25}$ was measured by ELISA (R&D, DANG20 for human ANGPT2) in the flow through with the standard of serial dilutions (10,000 pg/mL to 312.5 pg/mL) of recombinant $cANGPT2_{25}$ protein. Concentration of all isoforms of ANGPT2 was measured in intact serum or urine according to the manufacturer's instruction.

Human samples. ANGPT2 and its cleaved 25 kDa product were measured in serum. The samples of the intensive care unit group were collected within 24 h of ICU admission in patients with primary ARDS (n = 20), ARDS and sepsis (n = 10), and sepsis without ARDS (n = 10). Samples were immediately aliquoted and stored at -80°C until analyses. Clinical details are provided in **Table 2**. Serum was also collected from healthy controls (n = 12, mean age 30.9 ± 8.1, 50% female). The collection of the intensive care unit serum samples was approved according to the ethics committee of Hannover Medical School (Hannover, Germany) (MHH, EK #8146_BO_K_2018) and the collection of the control serum samples was approved by the institutional review board (IRB) of the University of Texas Southwestern Medical Center Dallas (USA). Urine was collected from a separate cohort of pediatric patients with sepsis and healthy controls (**Supplemental Table 7**). These samples were sent to the clinical laboratory at a tertiary care children’s hospital as part of routine care. Discarded clinical samples were collected for this study and stored at -80°C until measurement. The collection of these samples was approved by the UT Southwestern IRB.
Total ANGPT2 was measured using the ELISA from R&D following manufacturer’s instructions. For the analysis of the 25 kDa fragment of ANGPT2, a total of 100 µL of serum or 500 µL of urine

was applied to Amicon Ultra filters (50 kDa). Following centrifugation according to the manufacturer's instructions, concentrations of $cANGPT2_{25}$ were measured in the flow-through by ELISA using serial dilutions (10,000 pg/mL to 312.5 pg/mL) of recombinant $cANGPT_{25}$ protein to establish standard curves.

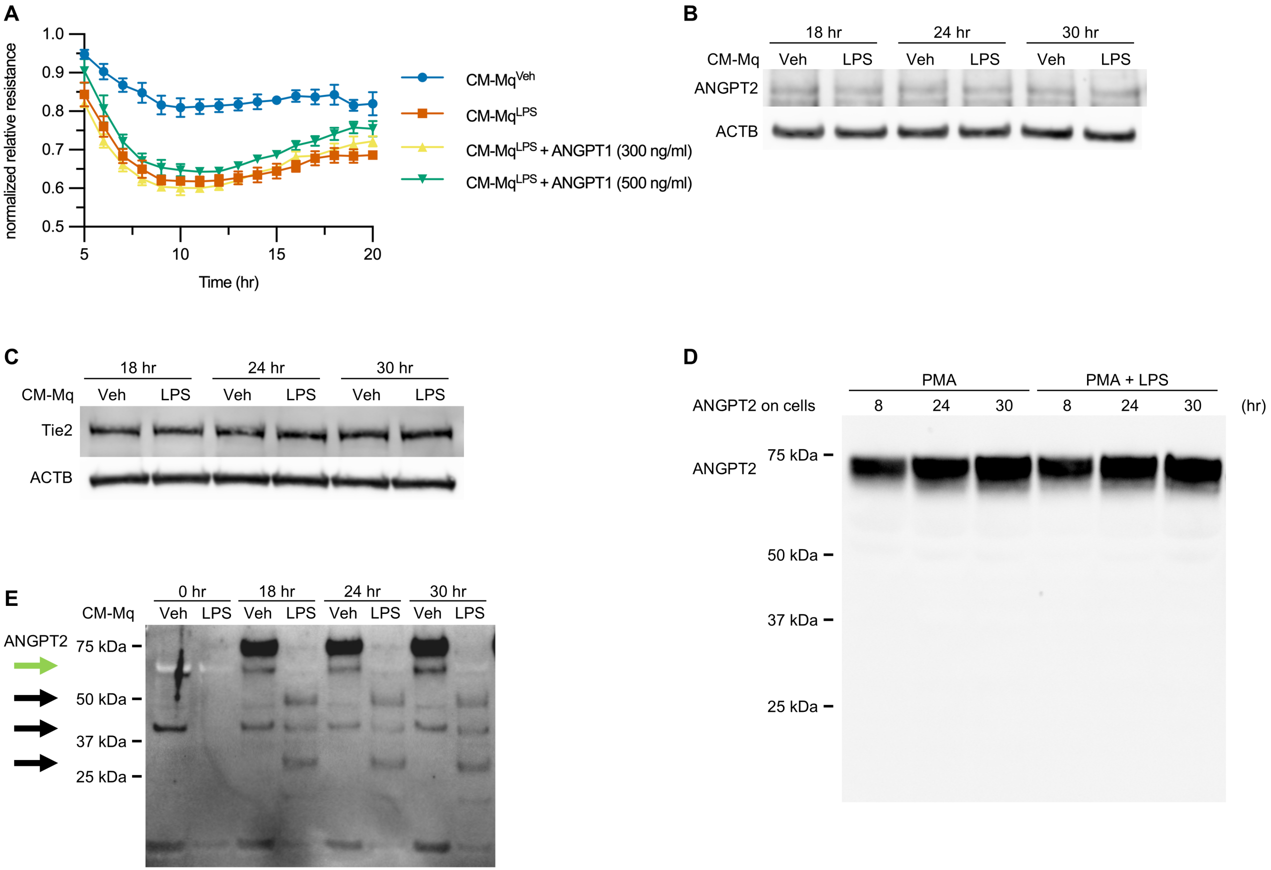

Supplemental Figure 1. Inflamed HUVECs do not autonomously process secreted ANGPT2. (A) Continuous recording of normalized monolayer electrical resistance with application of conditioned media from macrophages stimulated either with vehicle or LPS ($CM\text{-}Mq^{Veh}$ or $CM\text{-}Mq^{LPS}$) to confluent HUVEC monolayers with or without concomitant Angiopoietin-1 (ANGPT1) at the indicated concentration (n = 3 per group). (**B, C**) Western analysis of (**B**) ANGPT2 and (**C**) Tie2 in cell lysates of HUVECs incubated with CM-Mq for the indicated time. (**D**) Western analysis of ANGPT2 in CM of HUVECs under different activating conditions: phorbol ester (PMA)-differentiated HUVECs were incubated with recombinant ANGPT2 with or without LPS stimulation for the indicated time. (**E**) Uncropped image of Western analysis of ANGPT2 in HUVEC conditioned media. HUVECs were stimulated with $CM\text{-}Mq^{Veh}$ or $CM\text{-}Mq^{LPS}$ for the indicated time. Black arrows indicating bands specifically detected in CM of HUVEC stimulated with $CM\text{-}Mq^{LPS}$. The bands directly underneath ANGPT2 (green arrow) are presumed to be $ANGPT2_{443}$. Western analysis images are representative of at least three independent experiments.

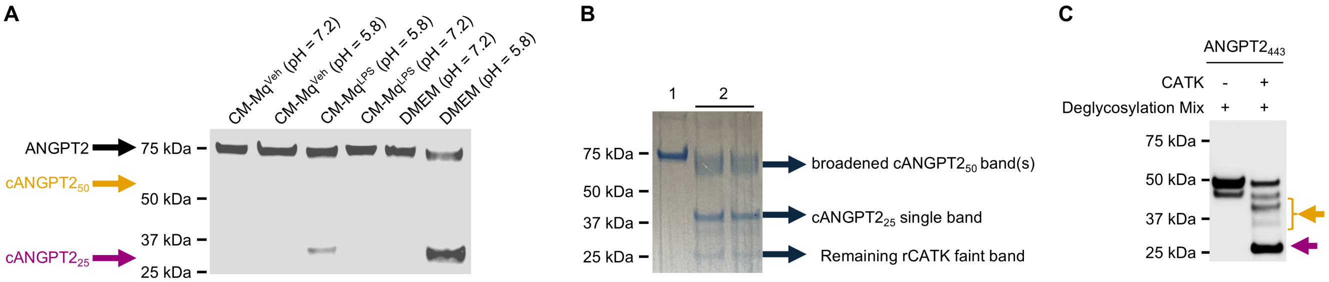

Supplemental Figure 2. Conditioned media of activated macrophages contains both a pH-dependent ANGPT2 cleaving activity and an inhibitor of cleavage activity. (A) Equal amounts of recombinant ANGPT2 and recombinant CATK were co-incubated in pH adjusted (7.2 or 5.8) CM-Mq^{Veh}, CM-Mq^{LPS}, or DMEM at 37°C for 20 minutes. Among the conditions, recombinant CATK is most efficient at cleaving ANGPT2 in DMEM at pH 5.8 (lane 6), an activity that is present but less efficient in the presence of CM-Mq^{LPS} at pH 5.8 (lane 3). (**B**) Coomassie Blue stained PVDF membrane after in vitro reaction of recombinant ANGPT2 and recombinant CATK: lane 1 contains 13 pmol of recombinant ANGPT2; lane 2 contains 160 pmol of recombinant ANGPT2 + 60 pmol of recombinant CATK. (**C**) Western analysis of HEK293 cells produced ANGPT2$_{443}$ treated with Deglycosylation Mix enzymes and CATK. Left arrows (yellow, purple) indicated deglycosylated cleaved ANGPT2$_{443}$ proteins. Western analysis images are representative of three independent experiments.

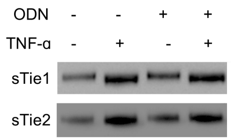

Supplemental Figure 3. ODN does not alter the cleavage of Tie1 and Tie2. Western analysis of Tie1 and Tie2 in CM of HUVECs. HUVECs pretreated with Veh (DMSO) or ODN for 60 minutes were stimulated with Veh (PBS) or TNF-α for 12 hours. Western analysis images are representative of three independent experiments.

A

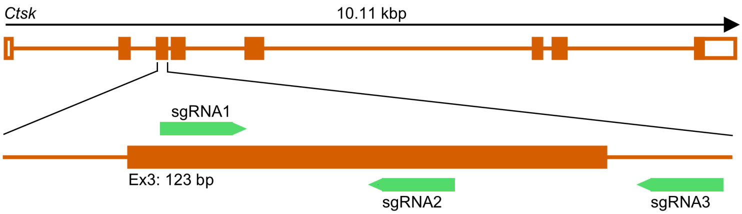

B

	Catk KO			Cas9 Cont.	
clone #	1	2	3	1	2
Indel %	100	100	100	0	0
KO score	96	100	95	-	-

C

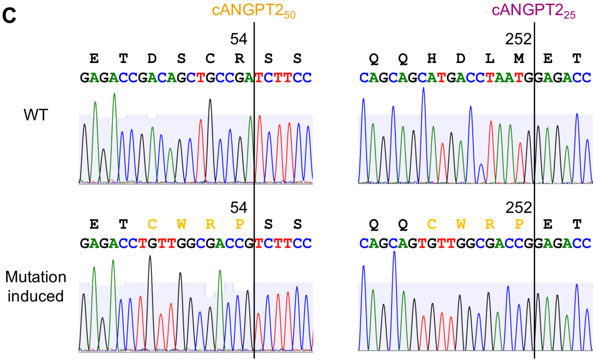

Supplemental Figure 4. Development of clonal Catk knockout RAW cell lines and uncleavable ANGPT2 vectors. (**A**) Strategy with 3 sgRNAs targeting the same region of *Ctsk* Ex3. Sequence of sgRNA1: AAAUCUCUCGGCGUUUAAUU; sgRNA2: GGACACCAAGAGAGGCCUCC; sgRNA3: UGGGCAGGCCUGGAUCUGAG. (**B**) Indel % and knockout (KO) score of *Ctsk* targeted cell lines. (**C**) Sanger sequencing chromatograms of Angpt2 expression vectors. Mutation induced at 51-54 aa (DSCR to CWRP) for cANGPT2$_{50}$ cleavage site, and at 249-252 aa (HDLM to CWRP) for cANGPT2$_{25}$ cleavage site.

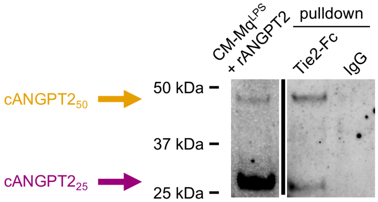

Supplemental Figure 5. Binding ability assay of cANGPT2s and Tie2. (**Left panel**) Recombinant ANGPT2 was transformed to cANGPT2$_{50}$ and cANGPT2$_{25}$ by addition of CM-Mq^{LPS}. (**Right panel**) CM-Mq^{LPS} containing cANGPT2$_{50}$ and cANGPT2$_{25}$ was then incubated with Tie2-Fc protein or control IgG protein to determine binding ability. Western analysis image is representative of three independent experiments.

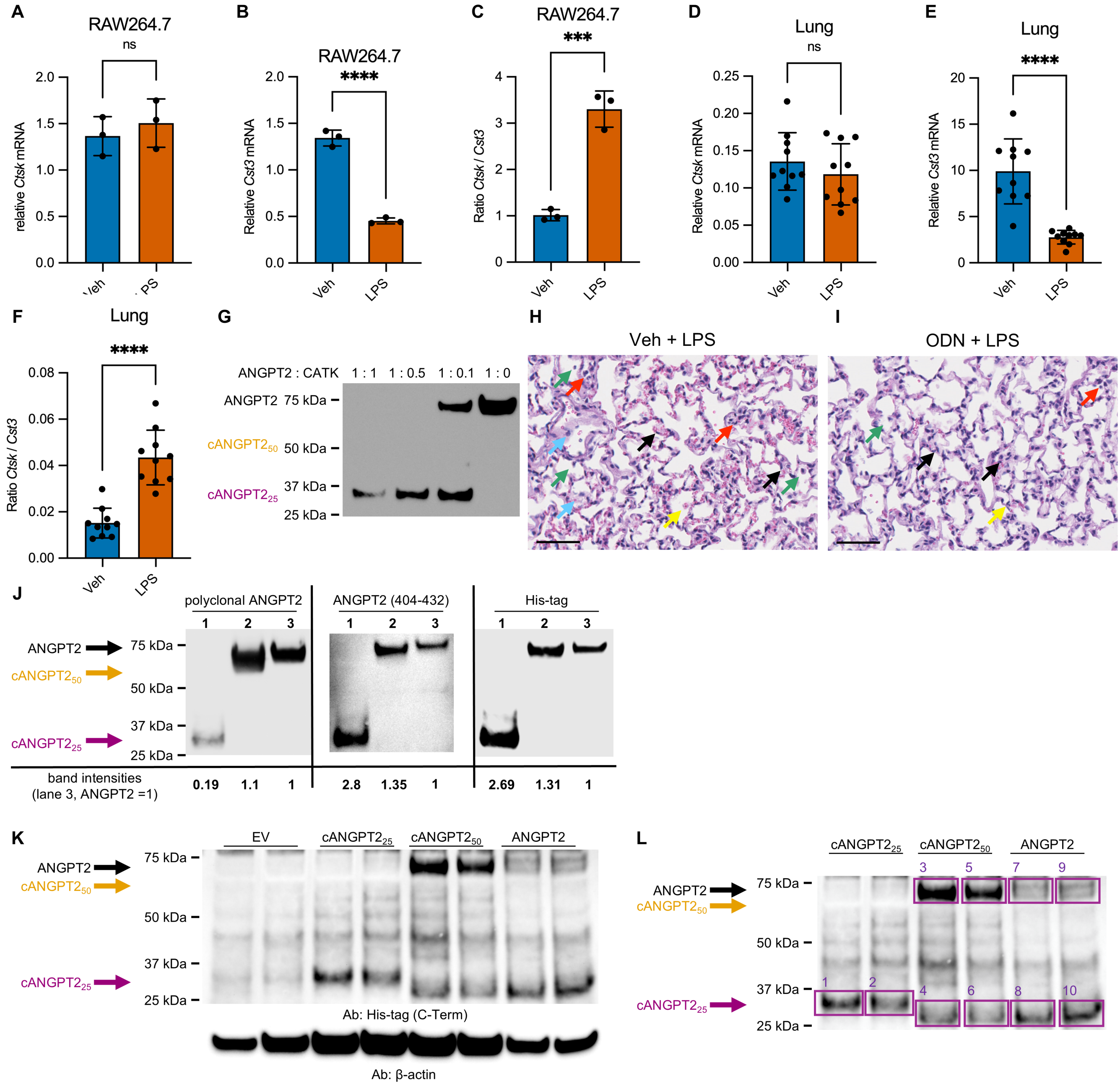

A
RAW264.7
ns
relative Ctsk mRNA
Veh
LPS
B
RAW264.7
****
Relative Cst3 mRNA
C
RAW264.7
***
Ratio Ctsk / Cst3
D
Lung
ns
Relative Ctsk mRNA
E
Lung
****
Relative Cst3 mRNA
F
Lung
****
Ratio Ctsk / Cst3
G
ANGPT2 : CATK 1 : 1 1 : 0.5 1 : 0.1 1 : 0
ANGPT2 75 kDa
cANGPT2₅₀ 50 kDa
cANGPT2₂₅ 37 kDa
25 kDa
H
Veh + LPS
I
ODN + LPS
J
polyclonal ANGPT2
ANGPT2 (404-432)
His-tag
1 2 3
ANGPT2
cANGPT2₅₀
cANGPT2₂₅
75 kDa
50 kDa
37 kDa
25 kDa
band intensities (lane 3, ANGPT2 =1)
0.19 1.1 1
2.8 1.35 1
2.69 1.31 1
K
EV
cANGPT2₂₅
cANGPT2₅₀
ANGPT2
Ab: His-tag (C-Term)
Ab: β-actin
L
cANGPT2₂₅
cANGPT2₅₀
ANGPT2
1 2 3 4 5 6 7 8 9 10

M

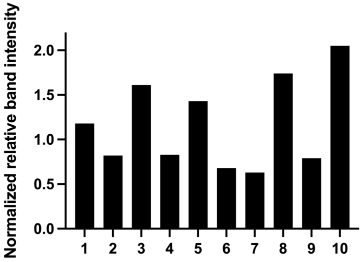

N

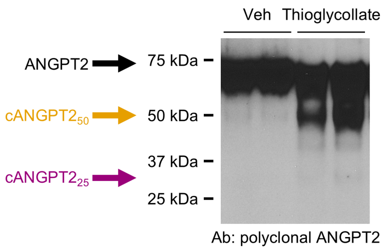

Supplemental Figure 6. Appearance and abundance of cleaved ANGPT2 in different models of inflammation. (**A, B**) Relative mRNA expression level of (**A**) Cathepsin K (*Ctsk)* and (**B**) Cystatin C (*Cst3)* in LPS stimulated RAW264.7 cells (n = 3 per group). (**C**) Ratio of *Ctsk/Cst3* mRNA level in RAW264.7 cells. (**D, E**) Relative mRNA expression level of (**D**) *Ctsk a*nd (**E**) *Cst3* in lung tissues of LPS administered mice (n = 10 mice per group). (**F**) Ratio of *Ctsk*/*Cst3* mRNA level in lung tissues. (**G**) Incubation of recombinant ANGPT2 and recombinant CATK. The ratio of ANGPT2 to CATK was varied from 1:1 to 1:0. (**H, I**) Representative images of H&E stained lung sections of (**H**) vehicle and LPS or (**I**) ODN (odanacatib, Cathepsin K inhibitor) and LPS treatment. Arrows indicate neutrophils in the alveolar space (green); neutrophils in the interstitial space (red); alveolar edema (light blue); proteinaceous debris filling the airspace (yellow); and alveolar septal thickening (black). Representative results of three animals per group were shown. Scale bar: 50 µm. (**J**) Western analysis with three different antibodies. Three identical membranes were made with CM of (1) cANGPT2$_{25}$, (2) cANGPT2$_{50}$, and (3) full-length ANGPT2 expression vector transfected HEK293 cells. Each membrane was blotted with polyclonal ANGPT2 Ab, ANGPT2 (404 -432) Ab, or Anti-His Ab as indicated. Quantification of band intensity relative to lane 3 which contains CM of HEK293 expressing full-length ANGPT2. Western analysis images of (**G**) and (**J**) are representative of three independent experiments. (**K**) Representative Western analysis of lung lysates of LPS administered mice. Mice received hydrodynamic tail vein gene: EV: control empty vector; cANGPT2$_{25}$; cANGPT2$_{50}$; or ANGPT2 (n = 2 per condition). Each expression vector contained 6x C-terminal His-tag. Vector delivery 4 hours prior to LPS administration. Lungs were harvested 24 hours post LPS administration. The membrane was blotted with anti-His antibody. (**L**) The image was cropped from (**K**), and each ANGPT2 band indicated by boxes 1 to 10 was quantified. (**M**) Quantification of band intensities of His-tagged full-length ANGPT2 and fragments from LPS-administered mouse lungs depicted in (**L**). (**N**) Incubation of peritoneal fluid with recombinant ANGPT2 at 37°C for 1 hour. Peritoneal fluid was harvested from vehicle (n = 2) or thioglycolate (n = 2) injected mice. The membrane was blotted with polyclonal ANGPT2 antibody. Relative band intensities of ANGPT2s. Each band intensity was first normalized to β-actin and then plotted as relative to average intensity of box 1 and 2. ***$p < 0.001$, ****$p < 0.0001$, ns, not significant by Mann-Whitney.

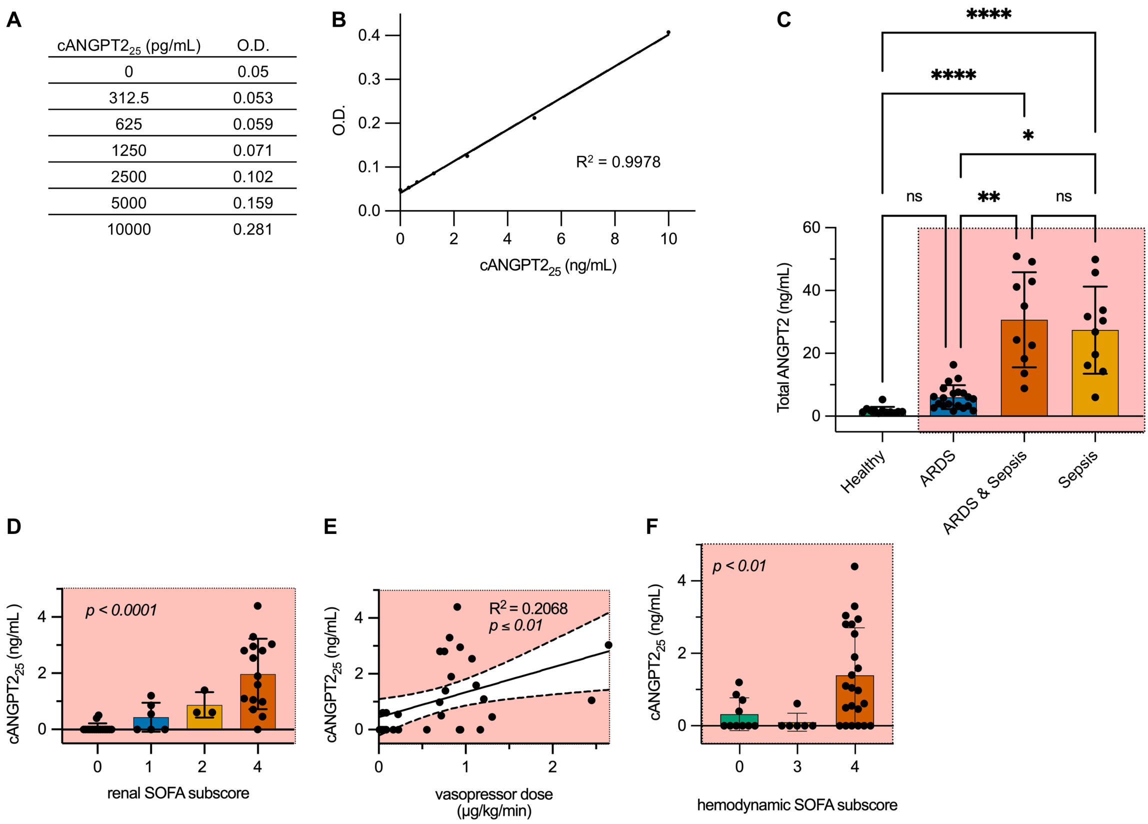

Supplemental Figure 7. Association of cleaved ANGPT2 with severity of critical illness in humans. (**A, B**) Representative standard curve of $cANGPT2_{25}$ ELISA. (**A**) Optical density (O.D.) of serial dilutions of recombinant $cANGPT2_{25}$ in manufacturer-provided diluent and (**B**) standard curve for $cANGPT2_{25}$ ELISA. (**C**) Bar graph showing the total amount of ANGPT2 (ng/mL) in serum of healthy controls (healthy, n = 10), patients in the intensive care unit with a primary diagnosis of acute respiratory distress syndrome (ARDS, n = 20), patients in the intensive care unit with a primary diagnosis of ARDS and sepsis (ARDS & Sepsis, n = 10) and patients in the intensive care unit with a primary diagnosis of sepsis (Sepsis, n = 10). Kruskal-Wallis, *ns* ≥ 0.051, *$p < 0.05$, **$p < 0.01$, ***$p < 0.001$. (**D**) Bar graph showing the amount of $cANGPT2_{25}$ (ng/mL) in sera of ICU patients with a primary diagnosis of ARDS, ARDS & Sepsis or Sepsis (n = 40) plotted against the renal SOFA sub score (1-4 points regarding creatinine concentration or urine output) (Kruskal-Wallis). (**E**) Scatter plot showing the positive linear regression of $cANGPT2_{25}$ (ng/mL) in sera of ICU patients with a diagnosis of ARDS, ARDS & Sepsis or Sepsis (n = 40) regarding the vasopressor dose received. (**F**) Bar graph showing the amount of $cANGPT2_{25}$ (ng/mL) in sera of ICU patients with a diagnosis of ARDS, ARDS & Sepsis or Sepsis (n = 40) plotted against the hemodynamic SOFA sub score (1-4 points regarding mean arterial pressure or vasopressor use (Kruskal Wallis).

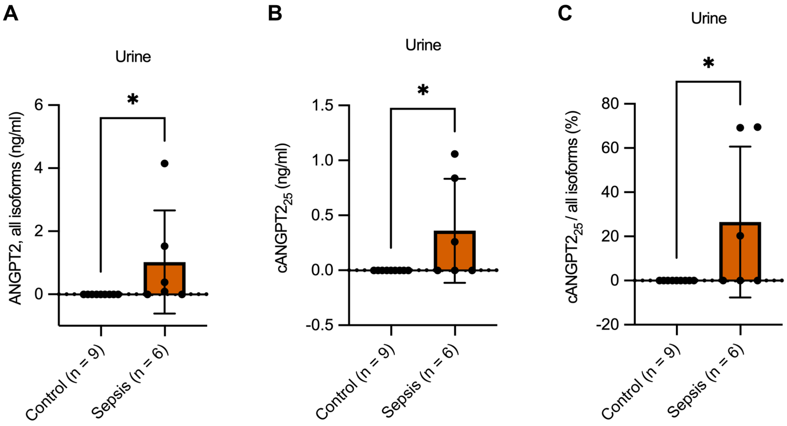

Supplemental Figure 8: Appearance of ANGPT2 fragments in urine of humans with sepsis. ELISA evaluation after size filtration of urine for (**A**) total ANGPT2 and (**B**) cANGPT2$_{25}$ in urine of sepsis patients (n = 6) and healthy volunteers (n = 9). (**C**) Ratio of cANGPT2$_{25}$ to all isoforms of ANGPT2. *$p < 0.05$ by Mann-Whitney.

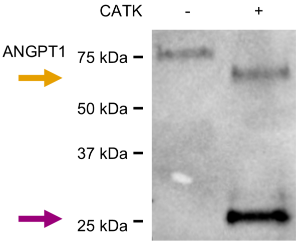

Supplemental Figure 9. CATK may cleave ANGPT1 in a similar manner to ANGPT2. Western analysis of recombinant ANGPT1 after incubation with recombinant CATK at 37°C for 5 minutes.

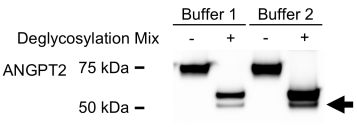

Supplemental Figure 10. Deglycosylation of recombinant ANGPT2. Deglycosylation of recombinant ANGPT2 produced doublet band (arrow). Western analysis image is representative of two independent experiments.

Supplemental Table 1. Sequences of primers for constructing ANGPT2 and cANGPT2s expression vectors.

Primer for	Forward/Reverse	Sequence (5’ → 3’)
ANGPT2	Forward	ttttctagagtttaaacaccatgtggcagattgttttctttac
ANGPT2	Reverse	tttgcggccgcgaattcttagaaatctgctggtcggatc
ANGPT2 with *IL2* signal peptide	Forward	gcattgcactaagtcttgcacttgtcacaaacagttataacaactttcggaagagc
cANGPT2$_{50}$	Forward	gcattgcactaagtcttgcacttgtcacaaacagttcttcctccagcccctacgtg
cANGPT2$_{25}$	Forward	gcattgcactaagtcttgcacttgtcacaaacagtgagacagttaataacttactg
Overhang extension PCR of all isoforms	Forward	cactggtaccaccatgtacaggatgcaactcctgtcttgcattgcactaagtc
Overhang extension PCR of all isoforms	Reverse	cgccgaattcttagtgatggtgatggtgatggaaatctgctggtcggatcatc

Supplemental Table 2. Primer sequences for RT-qPCR.

Gene	Forward Primer	Reverse Primer
ANGPT2	cagtggctaatgaagcttgaga	gttaacttccgcgtttgctc
HPRT1	cattatgctgaggatttggaaagg	cttgagcacacagagggctaca
Lcn2	atgtcacctccatcctggtcag	gccacttgcacattgtagctctg
Hprt1	acattgtggccctctgtgtg	ttatgtcccccgttgactga
Ctsk	ctcggcgtttaatttgggaga	ggaggtattctgagtcccgt
Cst3	gccatacaggtggtgagagc	gctggtcatggaaaggacag

Supplemental Table 3. MS analysis: Peptide sequences in reactions of ANGPT2 + CM-Mq^{Veh}, Asp-N fragmented

Peptide sequence	Modifications	Positions in ANGPT2
[M].DNCRSSSSPYVSNAVQR.[D]	1xCarbamidomethyl [C3]	51-67
[Y].DDSVQRLQVL.[E]		74-83
[Y].DDSVQRLQVLENIM.[E]		74-87
[D].DSVQRLQVL.[E]		75-83
[L].DQTSEINKLQ.[DN]		176-185
[Q].DKNSFLEKKVLAME.[D]		186-199
[E].DKHIIQLQSIK.[E]		200-210
[E].DKHIIQLQSIKEEK.[D]		200-213
[K].DQLQVLVSKQ.[N]		214-223
[K].DQLQVLVSKQNSIIEEL.[E]		214-230
[H].DLMETVNNL.[L]		250-258
[H].DLMETVNNLL.[T]		250-259
[H].DLMETVNNLL.[T]	1xOxidation [M3]	250-259
[H].DLMETVNNLLT.[M]		250-260
[H].DLMETVNNLLTM.[M]		250-261
[H].DLMETVNNLLTMM.[S]		250-262
[H].DLMETVNNLLTMMSTSNSAK.[D]		250-269
[H].DLMETVNNLLTMMSTSNSAK.[D]	1xOxidation [M]	250-269
[H].DLMETVNNLLTMMSTSNSAKDPTVAKEEQISFR.[D]		250-282
[M].MSTSNSAKDPTVAKEEQISFR.[D]		262-282
[S].TSNSAKDPTVAKEEQISFR.[D]		264-282
[K].DPTVAKEEQISFR.[D]		270-282
[D].PTVAKEEQISFR.[D]		271-282
[R].DCAEVFKSGHTT.[N]	1xCarbamidomethyl [C2]	283-294
[V].DFQRTWKEYKVGF.[G]		334-346
[K].DWEGNEAYSLYEHFYLSSEELNYR.[I]		377-400
[E].ELNYRIHLKGLTGTAGKISSISQPGN.[D]		396-421
[K].GLTGTAGKISSISQPGN.[D]		405-421
[G].TAGKISSISQPGN.[D]		409-421

Supplemental Table 4. MS analysis: Peptide sequences in reactions of ANGPT2 + CM-Mq^{LPS}, Asp-N fragmented

Peptide sequence	Modifications	Positions in ANGPT2
[A].YNNFRKSM.[D]		19-26
[A].YNNFRKSM.[D]	1xOxidation [M8]	19-26
[Y].NNFRKSM.[D]	1xOxidation [M7]	20-26
[M].DSIGKKQYQVQH.[G]		27-38
[Y].QVQHGSCSYTFLLPEM.[D]	1xCarbamidomethyl [C7]	35-50
[M].DNCRSSSSPYVSN.[A]	1xCarbamidomethyl [C3]	51-63
[M].DNCRSSSSPYVSNAVQR.[D]	1xCarbamidomethyl [C3]	51-67
[R].SSSSPYVSNAVQR.[D]		55-67
[S].SSSPYVSNAVQR.[D]		56-67
[S].SPYVSNAVQR.[D]		58-67
[R].DAPLEYDDSVQRLQVL.[E]		68-83
[Y].DDSVQRLQVL.[E]		74-83
[Y].DDSVQRLQVLENIM.[E]	1xOxidation [M14]	74-87
[D].DSVQRLQVL.[E]		75-83
[L].ENIMENNTQWLMKLENYIQ.[D]		84-102
[ML].ENNTQWLMKLENYIQ.[D]		88-102
[Q].DNMKKEMV.[E]	2xOxidation [M3; M7]	103-110
[Q].DNMKKEMVEIQQ.[N]		103-114
[A].EQTRKLT.[D]		137-143
[T].DVEAQVLNQTTRL.[E]		144-156
[L].ELQLLEHSLSTNKLEKQIL.[D]		157-175
[L].EHSLSTNKLEKQIL.[D]		162-175
[L].DQTSEINKLQ.[DN]		176-185
[Q].DKNSFLEKKVLAME.[D]		186-199
[Q].DKNSFLEKKVLAME.[D]	1xOxidation [M13]	186-199
[L].EKKVLAME.[D]		192-199
[L].EKKVLAME.[D]	1xOxidation [M7]	192-199
[E].DKHIIQLQSIK.[E]		200-210
[E].DKHIIQLQSIKEEK.[D]		200-213
[K].DQLQVLVSKQ.[N]		214-223
[K].DQLQVLVSKQN.[S]		214-224
[K].DQLQVLVSKQNSII.[E]		214-227
[K].DQLQVLVSKQNSIIE.[E]		214-228
[K].DQLQVLVSKQNSIIEEL.[E]		214-230
[K].DQLQVLVSKQNSIIEELEKKIVTA.[T]		214-237
[K].DQLQVLVSKQNSIIEELEKKIVTATVNNSVLQKQQH.[D]		214-249
[H].DLMETVNNL.[L]		250-258
[H].DLMETVNNLL.[T]		250-259
[H].DLMETVNNLL.[T]	1xOxidation [M3]	250-259
[H].DLMETVNNLLTMM.[S]		250-262
[H].DLMETVNNLLTMM.[S]	1xOxidation [M]	250-262
[H].DLMETVNNLLTMM.[S]	2xOxidation [M12; M13]	250-262
[H].DLMETVNNLLTMMSTS.[N]		250-265
[H].DLMETVNNLLTMMSTS.[N]	1xOxidation [M]	250-265
[H].DLMETVNNLLTMMSTS.[N]	2xOxidation [M12; M13]	250-265
[H].DLMETVNNLLTMMSTSN.[S]	1xOxidation [M]	250-266
[H].DLMETVNNLLTMMSTSNSAK.[D]		250-269
[H].DLMETVNNLLTMMSTSNSAK.[D]	1xOxidation [M]	250-269
[H].DLMETVNNLLTMMSTSNSAK.[D]	2xOxidation [M12; M]	250-269
[H].DLMETVNNLLTMMSTSNSAK.[D]	3xOxidation [M3; M12; M13]	250-269
[M].ETVNNLLTMMSTSNSAK.[D]		253-269
[M].ETVNNLLTMMSTSNSAK.[D]	1xOxidation [M9]	253-269
[L].LTMMSTSNSAK.[D]		259-269
[K].DPTVAKEEQISFR.[D]		270-282
[D].PTVAKEEQISFR.[D]		271-282
[P].TVAKEEQISFR.[D]		272-282
[T].VAKEEQISFR.[D]		273-282
[V].AKEEQISFR.[D]		274-282
[K].EEQISFR.[D]		276-282
[C].DMEAGGGGWTIIQRRE.[D]	1xOxidation [M2]	314-329
[V].DFQRTWKEYKVGFGNPSG.[E]		334-351
[K].DWEGNEAYSLYEHFYLSSEELNYR.[I]		377-400
[Y].LSSEELNYRIHLKGLTGTAGKISSISQPGN.[D]		392-421
[S].EELNYRIHLKGLTGTAGKISSISQPGN.[D]		395-421

[E].ELNYRIHLKGLTGTAGKISSISQPGN.[D]		396-421
[K].GLTGTAGKISSISQPGN.[D]		405-421
[N].DKCICKC.[S]	3xCarbamidomethyl [C3; C5; C7]	431-437
[N].DKCICKCS.[Q]	3xCarbamidomethyl [C3; C5; C7]	431-438
[N].DKCICKCSQMLTGGWWF.[D]	3xCarbamidomethyl [C3; C5; C7]	431-447

Supplemental Table 5. MS analysis: Peptide sequences in reactions of ANGPT2 + CM-Mq^{Veh}, trypsin fragmented

Peptide sequence	Modifications	Positions in ANGPT2
[V].LAAAYNNFRKSMDSIGKKQY.[Q]		15-34
[A].AAYNNFR.[K]		17-23
[M].DSIGKKQYQVQHG.[S]		27-39
[K].KQYQVQHGSCSY.[T]	1xCarbamidomethyl [C10]	32-43
[K].QYQVQHGSC.[S]	1xCarbamidomethyl [C9]	33-41
[K].QYQVQHGSCSY.[T]	1xCarbamidomethyl [C9]	33-43
[K].QYQVQHGSCSYTFLLPEMDNCR.[S]	2xCarbamidomethyl [C9; C21]	33-54
[K].QYQVQHGSCSYTFLLPEMDNCR.[S]	2xCarbamidomethyl [C9; C21]; 1xOxidation [M18]	33-54
[Y].QVQHGSCSYTFLLPEMDNCR.[S]	2xCarbamidomethyl [C7; C19]	35-54
[H].GSCSYTFLLPEMDNCR.[S]	2xCarbamidomethyl [C3; C15]	39-54
[G].SCSYTFLLPEMDNCR.[S]	2xCarbamidomethyl [C2; C14]	40-54
[C].SYTFLLPEMDNCR.[S]	1xCarbamidomethyl [C12]	42-54
[S].YTFLLPEMDNCR.[S]	1xCarbamidomethyl [C11]	43-54
[Y].TFLLPEMDNCR.[S]	1xCarbamidomethyl [C10]	44-54
[Y].TFLLPEMDNCR.[S]	1xCarbamidomethyl [C10]; 1xOxidation [M7]	44-54
[L].PEMDNCR.[S]	1xCarbamidomethyl [C6]	48-54
[N].CRSSSSPYVSNAVQRD.[A]	1xCarbamidomethyl [C1]	53-68
[R].SSSSPYVSN.[A]		55-63
[R].SSSSPYVSNAVQR.[D]		55-67
[S].SSSPYVSNAVQR.[D]		56-67
[S].SPYVSNAVQR.[D]		58-67
[S].PYVSNAVQR.[D]		59-67
[Y].VSNAVQR.[D]		61-67
[V].QRDAPLEYDDSVQRLQVLENIMENNTQWL.[M]		66-94
[Q].RDAPLEYDDSVQRLQVL.[E]		67-83
[R].DAPLEYDDSVQR.[L]		68-79
[R].DAPLEYDDSVQRLQVLENIMENNTQWLMKLENYIQDNM.[K]	1xOxidation [M20]	68-105
[D].APLEYDDSVQR.[L]		69-79
[A].PLEYDDSVQR.[L]		70-79
[R].LQVLENIMEN.[N]	1xOxidation [M8]	80-89
[L].QVLENIMENNTQ.[W]	1xOxidation [M7]	81-92
[V].LENIMENNTQWLMK.[L]	1xOxidation [M5]	83-96
[M].ENNTQWLMKLEN.[Y]		88-99
[T].QWLMKLENYIQDNMKKEMVE.[I]	2xOxidation [M14; M]	92-111
[Q].WLMKLENYIQDNMKKEMVEIQ.[Q]		93-113
[M].KLENYIQDNMK.[K]		96-106
[K].LENYIQDNMK.[K]		97-106
[K].LENYIQDNMK.[K]	1xOxidation [M9]	97-106
[E].NYIQDNMKKEMVEIQQ.[N]	1xOxidation [M]	99-114
[I].QDNMKKEMVEIQ.[Q]	1xOxidation [M8]	102-113
[Q].DNMKKEMVEI.[Q]	2xOxidation [M3; M7]	103-112
[Q].DNMKKEMVEIQQNAVQNQTAVMIE.[I]	1xOxidation [M22]	103-126
[D].NMKKEMVEI.[Q]	1xOxidation [M6]	104-112
[N].MKKEMVEIQQNAVQ.[N]		105-118
[M].KKEMVEIQQ.[N]	1xOxidation [M4]	106-114
[K].KEMVEIQQ.[N]	1xOxidation [M3]	107-114
[K].EMVEIQQN.[A]		108-115
[K].EMVEIQQN.[A]	1xOxidation [M2]	108-115
[E].MVEIQQNAVQNQ.[T]		109-120
[M].VEIQQNAVQNQTAVM.[I]	1xOxidation [M15]	110-124
[Q].NAVQNQTAVMIEIGTNLL.[N]	1xOxidation [M10]	115-132
[Q].NAVQNQTAVMIEIGTNLLNQ.[T]		115-134
[Q].NAVQNQTAVMIEIGTNLLNQ.[T]	1xOxidation [M10]	115-134
[V].QNQTAVMIEI.[G]		118-127
[V].QNQTAVMIEI.[G]	1xOxidation [M7]	118-127
[V].QNQTAVMIEIGTN.[L]		118-130
[Q].NQTAVMIEIGTNLLNQ.[T]	1xOxidation [M6]	119-134
[N].QTAVMIEIGTNLL.[N]		120-132
[N].QTAVMIEIGTNLLN.[Q]		120-133
[Q].TAVMIEIGTNLLNQ.[T]	1xOxidation [M4]	121-134
[A].VMIEIGTNLLNQ.[T]	1xOxidation [M2]	123-134
[M].IEIGTNLLNQ.[T]		125-134

[L].NQTAEQTRKLTDVEA.[Q]		133-147
[L].NQTAEQTRKLTDVEAQVLNQTTR.[L]		133-155
[T].AEQTRKLTDVEAQ.[V]		136-148
[R].KLTDVEAQVL.[N]		141-150
[R].KLTDVEAQVLNQ.[T]		141-152
[K].LTDVEAQVLNQTTR.[L]		142-155
[D].VEAQVLNQTTRL.[E]		145-156
[A].QVLNQTTRLE.[L]		148-157
[R].LELQLLEH.[S]		156-163
[R].LELQLLEHSL.[S]		156-165
[R].LELQLLEHSLSTN.[K]		156-168
[R].LELQLLEHSLSTNK.[L]		156-169
[L].ELQLLEHSLSTNK.[L]		157-169
[L].ELQLLEHSLSTNKL.[E]		157-170
[E].LQLLEHSLSTNK.[L]		158-169
[L].QLLEHSLSTNK.[L]		159-169
[Q].LLEHSLSTNK.[L]		160-169
[E].KQILDQTSEINK.[L]		172-183
[E].KQILDQTSEINKL.[Q]		172-184
[K].QILDQTSEINK.[L]		173-183
[K].QILDQTSEINKLQDK.[N]		173-187
[I].LDQTSEINKLQ.[D]		175-185
[D].QTSEINKLQDK.[N]		177-187
[Q].TSEINKLQDKNSFLEKKVLAM.[E]	1xOxidation [M21]	178-198
[S].EINKLQDKNSFLE.[K]		180-192
[K].KVLAMEDK.[H]		194-201
[K].KVLAMEDK.[H]	1xOxidation [M5]	194-201
[K].KVLAMEDKH.[I]	1xOxidation [M5]	194-202
[K].KVLAMEDKHII.[Q]		194-204
[K].KVLAMEDKHIIQL.[Q]	1xOxidation [M5]	194-206
[K].HIIQLQSIK.[E]		202-210
[K].HIIQLQSIKEEK.[D]		202-213
[H].IIQLQSIKEE.[K]		203-212
[L].QSIKEEKDQLQVL.[V]		207-219
[K].DQLQVLVSK.[Q]		214-222
[Q].LQVLVSKQNSIIEE.[L]		216-229
[L].QVLVSKQNSIIEEL.[E]		217-230
[K].QNSIIEELEK.[K]		223-232
[V].TATVNNSVLQKQQHD.[L]		236-250
[V].NNSVLQKQQHDLME.[T]		240-253
[Q].KQQHDLMETVNNLL.[T]		246-259
[K].QQHDLMETVNNLL.[T]		247-259
[K].QQHDLMETVNNLLT.[M]		247-260
[K].QQHDLMETVNNLLTM.[M]		247-261
[K].QQHDLMETVNNLLTM.[M]	1xOxidation [M15]	247-261
[K].QQHDLMETVNNLLTMM.[S]		247-262
[K].QQHDLMETVNNLLTMMSTSNSAK.[D]		247-269
[K].QQHDLMETVNNLLTMMSTSNSAK.[D]	1xOxidation [M]	247-269
[K].QQHDLMETVNNLLTMMSTSNSAK.[D]	2xOxidation [M15; M16]	247-269
[H].DLMETVNNLLTM.[M]		250-261
[H].DLMETVNNLLTMM.[S]		250-262
[H].DLMETVNNLLTMMSTSNSAK.[D]		250-269
[D].LMETVNNLLT.[M]		251-260
[E].TVNNLLTMMSTSNSAKDPTVA.[K]		254-274
[E].TVNNLLTMMSTSNSAKDPTVAK.[E]	2xOxidation [M8; M9]	254-275
[N].NLLTMMSTSNSAKDPTVAKEEQISFRDCA.[E]	1xCarbamidomethyl [C28]; 1xOxidation [M6]	257-285
[N].LLTMMSTSNSAKDPTVAKEEQISF.[R]		258-281
[T].SNSAKDPTVAKEEQI.[S]		265-279
[K].EEQISFR.[D]		276-282
[E].EQISFRDCAEVFKSGHTTNGIYTLTFPNSTE.[E]	1xCarbamidomethyl [C8]	277-307
[R].DCAEVFK.[S]	1xCarbamidomethyl [C2]	283-289
[Y].TLTFPNSTEEIKAYCDMEAGGGGW.[T]	1xCarbamidomethyl [C15]	299-322
[L].TFPNSTEEIKAYCDMEAGGGGW.[T]	1xCarbamidomethyl [C13]; 1xOxidation [M15]	301-322
[S].TEEIKAYCDMEAGGGGWTIIQ.[R]	1xCarbamidomethyl [C8]	306-326

[K].AYCDMEAGGGGWTIIQR.[R]	1xCarbamidomethyl [C3]	311-327
[K].AYCDMEAGGGGWTIIQR.[R]	1xCarbamidomethyl [C3]; 1xOxidation [M5]	311-327
[D].MEAGGGGWTIIQRREDGSVDFQRTW.[K]		315-339
[M].EAGGGGWTIIQ.[R]		316-326
[M].EAGGGGWTIIQRR.[E]		316-328
[M].EAGGGGWTIIQRRE.[D]		316-329
[E].AGGGGWTIIQRRE.[D]		317-329
[E].AGGGGWTIIQRRED.[G]		317-330
[R].REDGSVDFQR.[T]		328-337
[R].REDGSVDFQRTWK.[E]		328-340
[R].EDGSVDFQR.[T]		329-337
[K].VGFGNPSGEY.[W]		344-353
[K].VGFGNPSGEYWLGN.[E]		344-357
[K].VGFGNPSGEYWLGNEF.[V]		344-359
[K].VGFGNPSGEYWLGNEFVSQL.[T]		344-363
[K].VGFGNPSGEYWLGNEFVSQLTN.[Q]		344-365
[K].VGFGNPSGEYWLGNEFVSQLTNQQR.[Y]		344-368
[F].GNPSGEYWLGNEFVSQLTNQQR.[Y]		347-368
[N].PSGEYWLGNEFVSQLTNQQR.[Y]		349-368
[Y].WLGNEFVSQLTNQQR.[Y]		354-368
[W].LGNEFVSQLTNQQR.[Y]		355-368
[N].EFVSQLTNQQR.[Y]		358-368
[F].VSQLTNQQR.[Y]		360-368
[K].DWEGNEAYSLYEHF.[Y]		377-390
[H].FYLSSEELNYR.[I]		390-400
[F].YLSSEELNYR.[I]		391-400
[Y].LSSEELNYR.[I]		392-400
[E].LNYRIHLKGLTGTA.[G]		397-410
[L].TGTAGKISSISQPGN.[D]		407-421
[G].TAGKISSISQPGNDFSTKDGDNDK.[C]		409-432
[K].ISSISQPGNDFSTK.[D]		413-426
[S].SISQPGNDFSTK.[D]		415-426
[S].ISQPGNDFSTK.[D]		416-426
[M].LTGGWWFDACGPSN.[L]	1xCarbamidomethyl [C10]	441-454
[L].TGGWWFDACGPSNLNGMYYPQR.[Q]	1xCarbamidomethyl [C9]	442-463
[T].GGWWFDACGPSNLNGMYYPQR.[Q]	1xCarbamidomethyl [C8]	443-463
[G].GWWFDACGP.[S]	1xCarbamidomethyl [C7]	444-452
[W].WFDACGPSNLNGMYYPQR.[Q]	1xCarbamidomethyl [C5]	446-463
[W].WFDACGPSNLNGMYYPQR.[Q]	1xCarbamidomethyl [C5]; 1xOxidation [M13]	446-463
[F].DACGPSNLNGMYYPQR.[Q]	1xCarbamidomethyl [C3]	448-463
[C].GPSNLNGMYYPQR.[Q]		451-463
[T].NKFNGIKWYYWKGSGYSLKATTMMIRPA.[D]		467-494
[F].NGIKWYYWKGSGYSL.[K]		470-484
[W].YYWKGSGYSLKATTMMIRPADF.[-]	1xOxidation [M15]	475-496
[L].KATTMMIRP.[A]	1xOxidation [M6]	485-493

Supplemental Table 6. MS analysis: Peptide sequences in reactions of ANGPT2 + CM-Mq^{LPS}, trypsin fragmented

Peptide sequence	Modifications	Positions in ANGPT2
[F].TLSCDLVL.[A]	1xCarbamidomethyl [C4]	8-15
[Y].NNFRKSMDSI.[G]	1xOxidation [M7]	20-29
[R].KSMDSIGKK.[Q]	1xOxidation [M3]	24-32
[K].SMDSIGK.[K]		25-31
[K].QYQVQHGSCSY.[T]	1xCarbamidomethyl [C9]	33-43
[Q].YQVQHGSCSY.[T]	1xCarbamidomethyl [C8]	34-43
[Q].VQHGSCSY.[T]	1xCarbamidomethyl [C6]	36-43
[C].RSSSSPYVSNAVQ.[R]		54-66
[R].SSSSPYVSNAVQR.[D]		55-67
[S].SSSPYVSNAVQR.[D]		56-67
[S].SSPYVSNAVQR.[D]		57-67
[S].PYVSNAVQR.[D]		59-67
[Y].VSNAVQR.[D]		61-67
[R].DAPLEYDDSVQR.[L]		68-79
[A].PLEYDDSVQR.[L]		70-79
[A].PLEYDDSVQRLQ.[V]		70-81
[M].ENNTQWLMK.[L]		88-96
[M].ENNTQWLMK.[L]	1xOxidation [M8]	88-96
[M].ENNTQWLMKL.[E]	1xOxidation [M8]	88-97
[E].NNTQWLMKLE.[N]		89-98
[W].LMKLENYI.[Q]	1xOxidation [M2]	94-101
[K].LENYIQDN.[M]		97-104
[K].LENYIQDNMK.[K]		97-106
[K].LENYIQDNMK.[K]	1xOxidation [M9]	97-106
[I].QDNMKKEMVEI.[Q]		102-112
[I].QDNMKKEMVEI.[Q]	2xOxidation [M4; M8]	102-112
[K].KEMVEIQQN.[A]		107-115
[K].EMVEIQQN.[A]		108-115
[E].MVEIQQNAVQNQ.[T]		109-120
[Q].QNAVQNQTAVMIEI.[G]		114-127
[Q].NAVQNQTAVMI.[E]		115-125
[V].QNQTAVMIEIGTN.[L]	1xOxidation [M7]	118-130
[Q].NQTAVMIEIGTN.[L]		119-130
[T].AVMIEIGTNLLNQ.[T]		122-134
[E].IGTNLLNQ.[T]		127-134
[R].KLTDVEAQVLNQ.[T]		141-152
[V].EAQVLNQTTRLE.[L]		146-157
[Q].VLNQTTRLELQL.[L]		149-160
[R].LELQLLE.[H]		156-162
[R].LELQLLEH.[S]		156-163
[R].LELQLLEHSLSTNK.[L]		156-169
[L].QLLEHSLSTNK.[L]		159-169
[K].QILDQTSEINK.[L]		173-183
[Q].ILDQTSEINK.[L]		174-183
[I].LDQTSEINK.[L]		175-183
[L].DQTSEINK.[L]		176-183
[L].QDKNSFLEK.[K]		185-193
[K].KVLAMEDKH.[I]	1xOxidation [M5]	194-202
[K].KVLAMEDKHIIQL.[Q]	1xOxidation [M5]	194-206
[K].VLAMEDKHIIQLQSIKEEKDQL.[Q]	1xOxidation [M4]	195-216
[K].HIIQLQSIK.[E]		202-210
[S].IKEEKDQLQ.[V]		209-217
[K].DQLQVLVSK.[Q]		214-222
[K].QQHDLMETVNNL.[L]		247-258
[K].QQHDLMETVNNLL.[T]	1xOxidation [M6]	247-259
[M].ETVNNLLT.[M]		253-260
[M].ETVNNLLTM.[M]		253-261
[M].ETVNNLLTM.[M]	1xOxidation [M9]	253-261
[M].ETVNNLLTMM.[S]		253-262
[M].ETVNNLLTMM.[S]	1xOxidation [M]	253-262
[M].ETVNNLLTMMSTSN.[S]		253-266
[M].ETVNNLLTMMSTSN.[S]	1xOxidation [M]	253-266
[E].TVNNLLTMMSTSNSAKDPTVAK.[E]		254-275
[N].NLLTMMSTSNSAKDPTVAK.[E]		257-275

[N].LLTMMSTSNSAKDP.[T]	1xOxidation [M4]	258-271
[T].MMSTSNSAK.[D]	1xOxidation [M1]	261-269
[T].SNSAKDPTVAKEEQI.[S]		265-279
[A].KDPTVAK.[E]		269-275
[K].EEQISFR.[D]		276-282
[R].DCAEVFK.[S]	1xCarbamidomethyl [C2]	283-289
[D].CAEVFK.[S]	1xCarbamidomethyl [C1]	284-289
[K].SGHTTNGIYT.[L]		290-299
[K].SGHTTNGIYTLT.[F]		290-301
[T].LTFPNSTEEI.[K]		300-309
[T].FPNSTEEIK.[A]		302-310
[E].IKAYCDMEAGGG.[G]	1xCarbamidomethyl [C5]	309-320
[K].AYCDMEAGGGGW.[T]	1xCarbamidomethyl [C3]	311-322
[K].AYCDMEAGGGGWTIIQR.[R]	1xCarbamidomethyl [C3]	311-327
[K].AYCDMEAGGGGWTIIQR.[R]	1xCarbamidomethyl [C3];<br>1xOxidation [M5]	311-327
[Y].CDMEAGGGGWTIIQR.[R]	1xCarbamidomethyl [C1]	313-327
[D].MEAGGGGWTIIQR.[R]		315-327
[M].EAGGGGWTII.[Q]		316-325
[M].EAGGGGWTIIQ.[R]		316-326
[M].EAGGGGWTIIQR.[R]		316-327
[E].AGGGGWTIIQ.[R]		317-326
[E].AGGGGWTIIQR.[R]		317-327
[E].AGGGGWTIIQRRE.[D]		317-329
[R].REDGSVDFQR.[T]		328-337
[R].EDGSVDFQR.[T]		329-337
[R].EDGSVDFQRTW.[K]		329-339
[R].EDGSVDFQRTWKE.[Y]		329-341
[E].DGSVDFQR.[T]		330-337
[K].VGFGNPSGEYWLGNEFVSQ.[L]		344-362
[K].VGFGNPSGEYWLGNEFVSQL.[T]		344-363
[K].VGFGNPSGEYWLGNEFVSQLT.[N]		344-364
[K].VGFGNPSGEYWLGNEFVSQLTNQQR.[Y]		344-368
[G].FGNPSGEYWLGNEFVSQLTNQQR.[Y]		346-368
[F].GNPSGEYWLGNEFVSQL.[T]		347-363
[F].GNPSGEYWLGNEFVSQLTN.[Q]		347-365
[F].GNPSGEYWLGNEFVSQLTNQQR.[Y]		347-368
[G].NPSGEYWLGNEFVSQL.[T]		348-363
[G].NPSGEYWLGNEFVSQLTN.[Q]		348-365
[G].NPSGEYWLGNEFVSQLTNQQR.[Y]		348-368
[N].PSGEYWLGNEFVSQLTNQQR.[Y]		349-368
[P].SGEYWLGNEFVSQLTNQQR.[Y]		350-368
[G].EYWLGNEFVSQLTNQQR.[Y]		352-368
[Y].WLGNEFVSQLTNQQR.[Y]		354-368
[W].LGNEFVSQLTNQQR.[Y]		355-368
[L].GNEFVSQLTNQQR.[Y]		356-368
[G].NEFVSQLTNQQR.[Y]		357-368
[N].EFVSQLTNQQR.[Y]		358-368
[E].FVSQLTNQQR.[Y]		359-368
[F].VSQLTNQQR.[Y]		360-368
[S].QLTNQQR.[Y]		362-368
[K].DWEGNEAYSL.[Y]		377-386
[K].DWEGNEAYSLY.[E]		377-387
[K].DWEGNEAYSLYEH.[F]		377-389
[K].DWEGNEAYSLYEHF.[Y]		377-390
[K].DWEGNEAYSLYEHFY.[L]		377-391
[K].DWEGNEAYSLYEHFYLSSEELNYR.[I]		377-400
[Y].SLYEHFYLSSEELNYR.[I]		385-400
[H].FYLSSEELNYR.[I]		390-400
[F].YLSSEELNYR.[I]		391-400
[Y].LSSEELNYR.[I]		392-400
[L].SSEELNYR.[I]		393-400
[S].SEELNYR.[I]		394-400
[R].IHLKGLTGTAGK.[I]		401-412
[L].KGLTGTAGK.[I]		404-412
[T].GTAGKISSISQPGNDFSTK.[D]		408-426

[G].TAGKISSISQPGNDFSTK.[D]		409-426
[T].AGKISSISQPGNDFSTK.[D]		410-426
[K].ISSISQPGNDF.[S]		413-423
[K].ISSISQPGNDFSTK.[D]		413-426
[K].ISSISQPGNDFSTKD.[G]		413-427
[I].SSISQPGNDFSTK.[D]		414-426
[S].SISQPGNDFSTK.[D]		415-426
[S].ISQPGNDFSTK.[D]		416-426
[I].SQPGNDFSTKDGDNDK.[C]		417-432
[L].TGGWWFDACGPSNLNGMYYPQR.[Q]	1xCarbamidomethyl [C9]	442-463
[L].TGGWWFDACGPSNLNGMYYPQR.[Q]	1xCarbamidomethyl [C9]; 1xOxidation [M17]	442-463
[T].GGWWFDACGPSNLNGMYYPQR.[Q]	1xCarbamidomethyl [C8]	443-463
[T].GGWWFDACGPSNLNGMYYPQR.[Q]	1xCarbamidomethyl [C8]; 1xOxidation [M16]	443-463
[W].WFDACGPSNLNGMYYPQR.[Q]	1xCarbamidomethyl [C5]	446-463
[F].DACGPSNLNGMYYPQR.[Q]	1xCarbamidomethyl [C3]	448-463
[F].DACGPSNLNGMYYPQR.[Q]	1xCarbamidomethyl [C3]; 1xOxidation [M11]	448-463
[D].ACGPSNLNGMYYPQR.[Q]	1xCarbamidomethyl [C2]	449-463
[D].ACGPSNLNGMYYPQR.[Q]	1xCarbamidomethyl [C2]; 1xOxidation [M10]	449-463
[A].CGPSNLNGMYYPQR.[Q]	1xCarbamidomethyl [C1]	450-463
[C].GPSNLNGMYYPQR.[Q]		451-463
[C].GPSNLNGMYYPQR.[Q]	1xOxidation [M8]	451-463
[G].PSNLNGMYYPQR.[Q]		452-463
[S].NLNGMYYPQR.[Q]		454-463
[N].LNGMYYPQR.[Q]		455-463
[L].NGMYYPQR.[Q]		456-463
[N].TNKFNGIKWYYWKGSGYSLKAT.[T]		466-487
[K].ATTMMIR.[P]		486-492
[K].ATTMMIR.[P]	1xOxidation [M]	486-492

Supplemental Table 7. Characteristics of patients tested for presence of urinary $cANGPT2_{25}$.

	Septic Patients (n = 6)	Controls (n = 9)	*p* value
Age (Mean [SD])	10.9 [7.70]	11.4 [5.11]	0.85
Female (%)	63.6	35.0	0.13
Hispanic (%)	27.3	55.0	0.13
White (%)	36.3	50.0	0.53
Bacterial sepsis (%)	81.8		
COVID sepsis (%)	18.2		
ECMO (%)	36.4		
CRRT (%)	18.2		

Supplemental Table 8. List of antibodies.

Antibody	Host	Supplier	Number
polyclonal C-terminal ANGPT2	Goat	R&D	AF623
Phospho-AKT (ser473)	Rabbit	CST	9271
AKT	Rabbit	CST	9272
β-Actin (13E5)	Rabbit	CST	5125
TIE1	Rabbit	Genetex	GTX103429
TIE2	Mouse	Millipore	05-584
ANGPT2 (404-432)	Rabbit	Invitrogen	PA5-23612
His-tag	Mouse	R&D	MAB050
His-tag (C-Term)	Rabbit	GenScript	A01857
ANGPT1	Rabbit	abcam	ab183702
