# Supplementary material for: Cathepsin K cleavage of angiopoietin-2 creates detrimental Tie2 antagonist fragments in sepsis
Source: J Clin Invest. 2025 Mar 3;135(8):e174135. doi: 10.1172/JCI174135 (PMC11996858; doi:10.1172/JCI174135)

Full unedited membrane for Figure 1E

lanes used for the figure

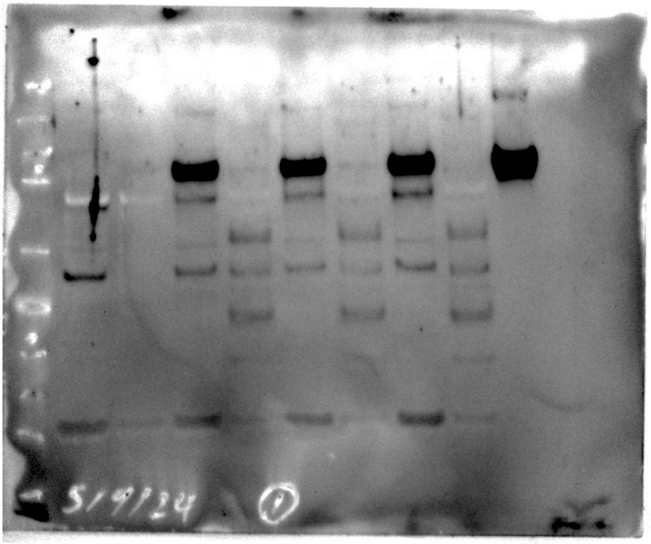

Full unedited film for Figure 1G

lanes used for the figure

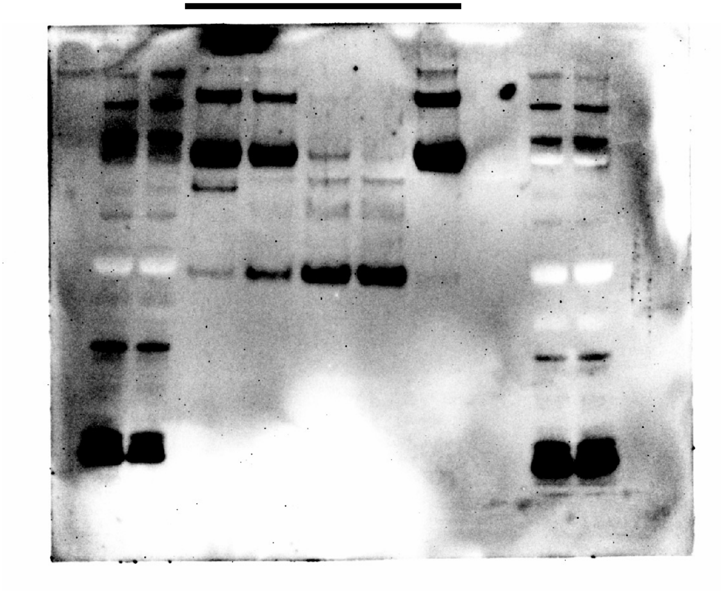

Full unedited membrane for Figure 1H

lanes used for the figure

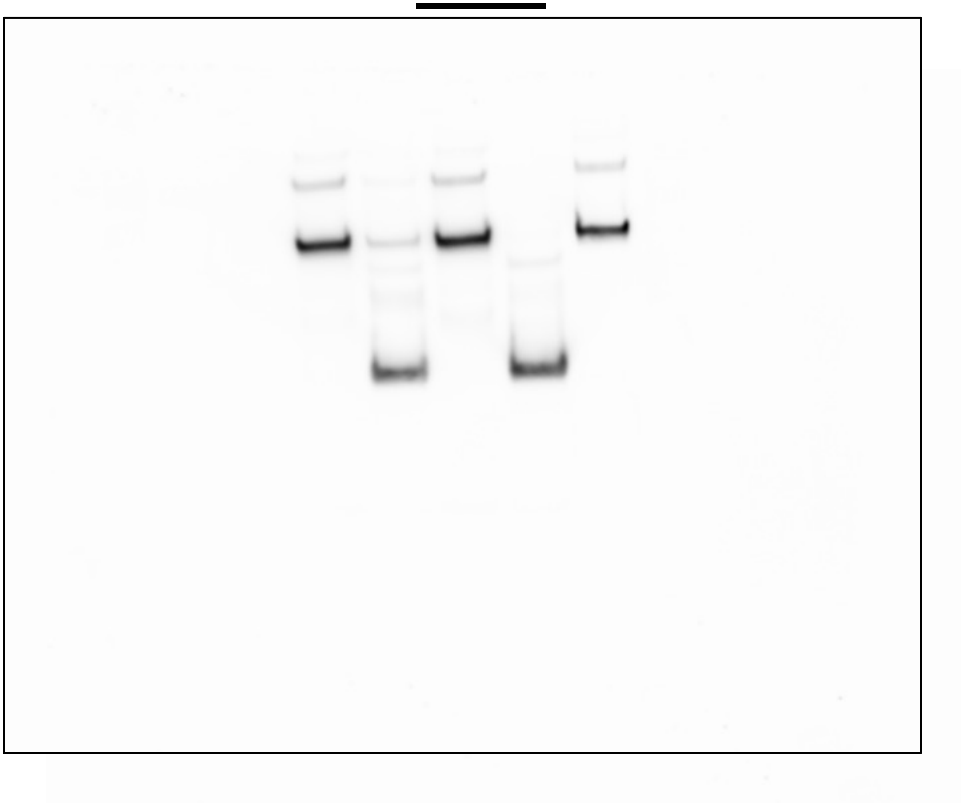

Full unedited membrane for Figure 2A

lane used for the figure

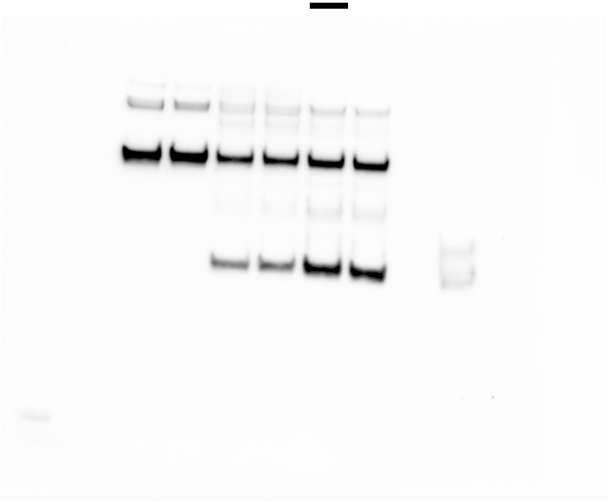

lane used for the figure

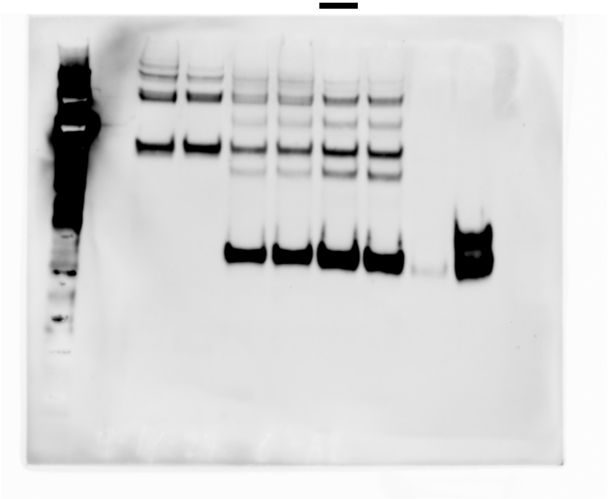

Full unedited membrane for Figure 2B

lanes used for the figure

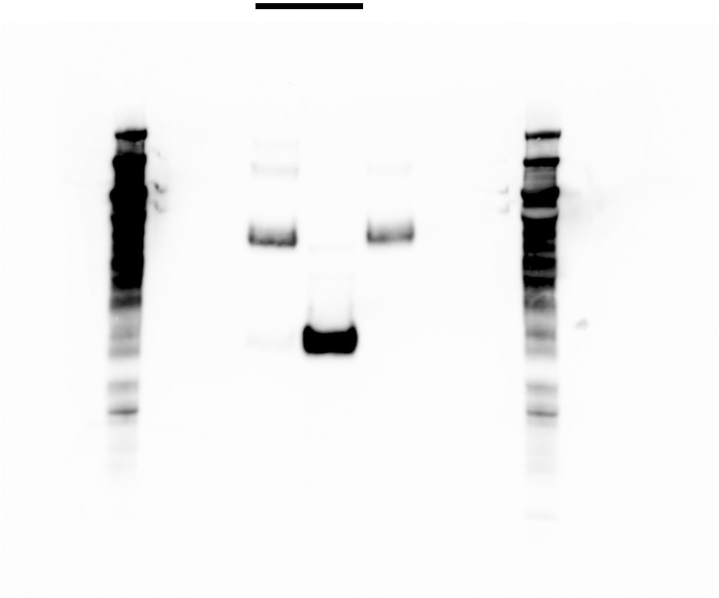

Full unedited film for Figure 2D

lanes used for the figure

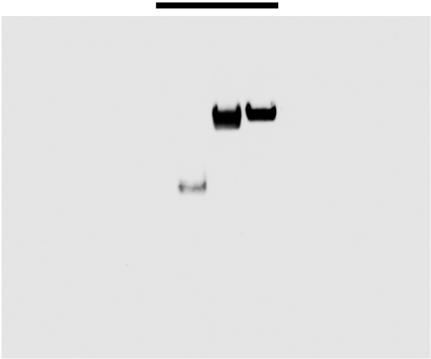

This Figure is used in Supplemental
Figure 6J, C'-ANGPT2 lanes, too

Full unedited film for Figure 2G

lanes used for the figure,
ANGPT2 left two lanes

lanes used for the figure,
ANGPT2 right two lanes

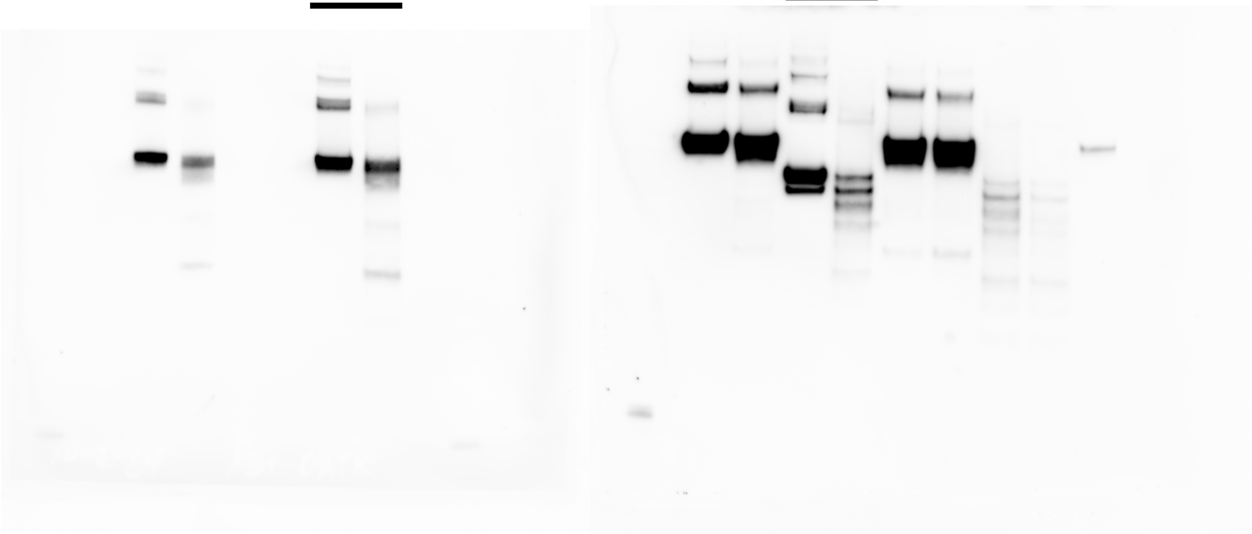

Full unedited film for Figure 2G

lanes used for the figure, ANGPT2 (55S-496F) left two lanes

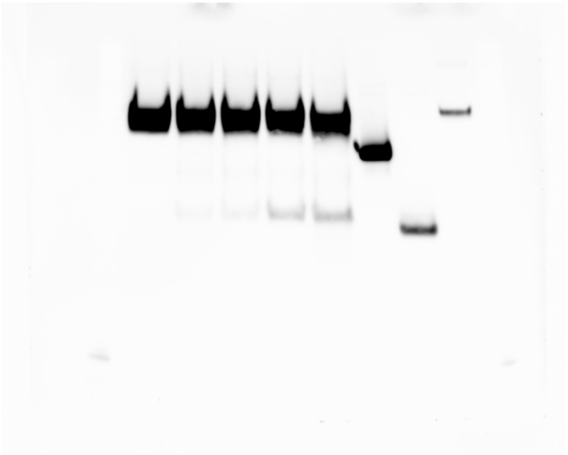

lanes used for the figure, ANGPT2 (55S-496F) right two lanes

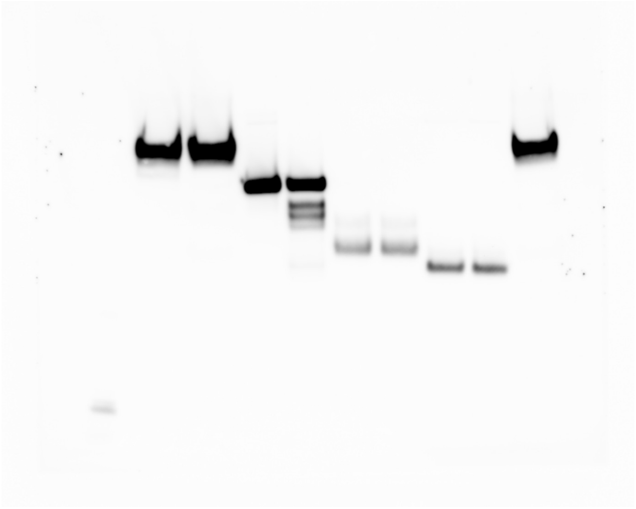

Full unedited film for Figure 2G

lanes used for the figure, ANGPT2
(253E-496F)

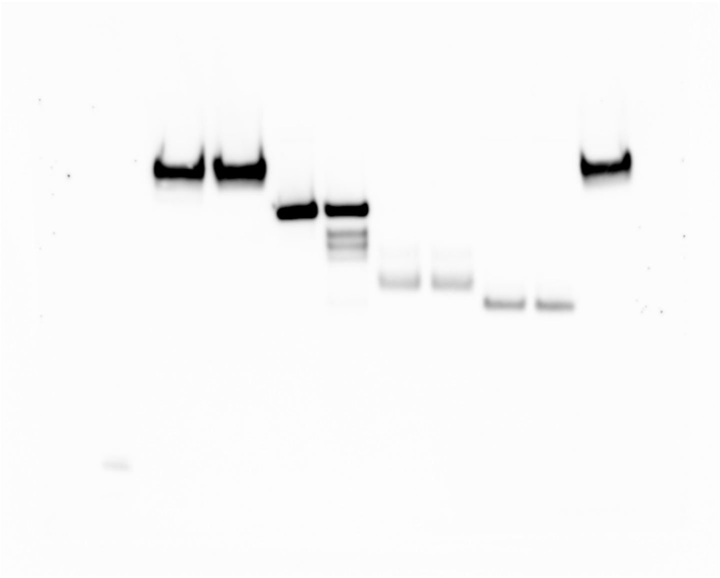

Full unedited membrane for Figure 3A

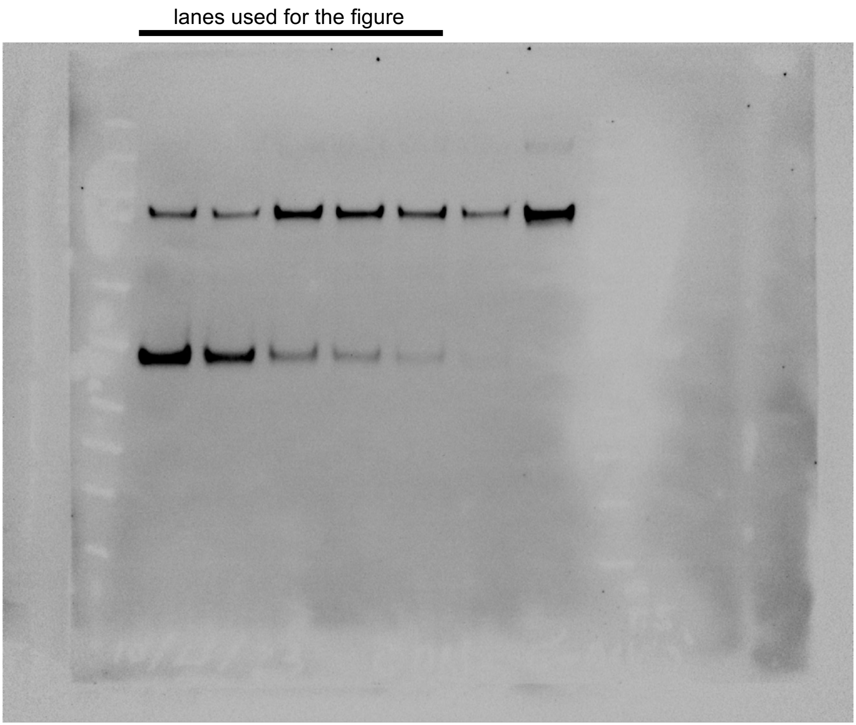

Full unedited film for Figure 3B

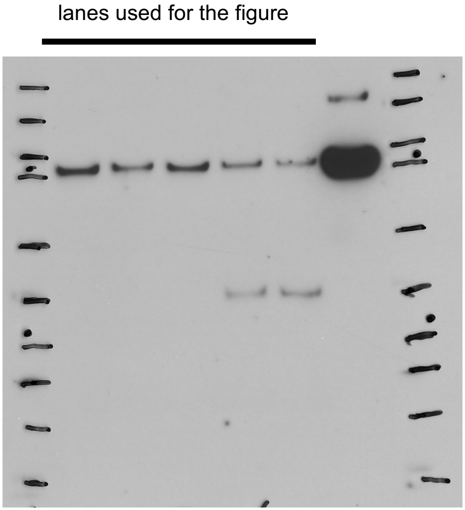

Full unedited membrane for Figure 3C

lanes used for the figure

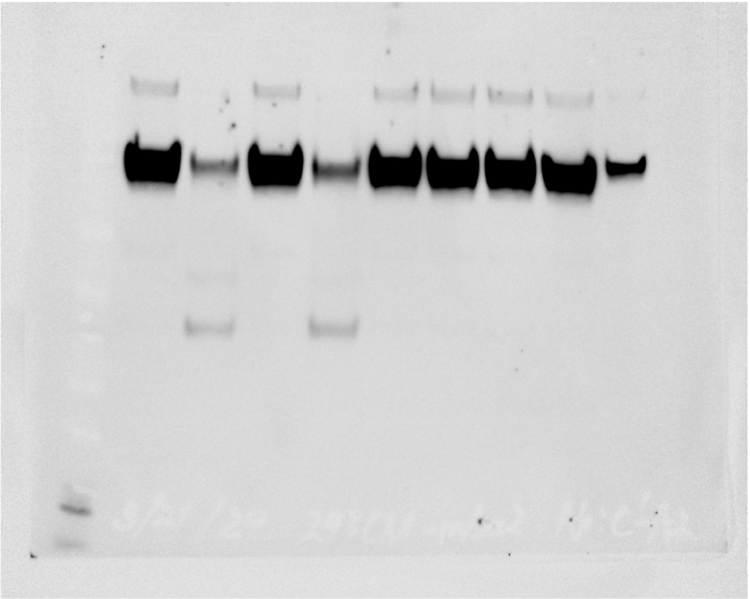

Full unedited filml for Figure 3D

lanes used for the figure, left side, Reduced condition

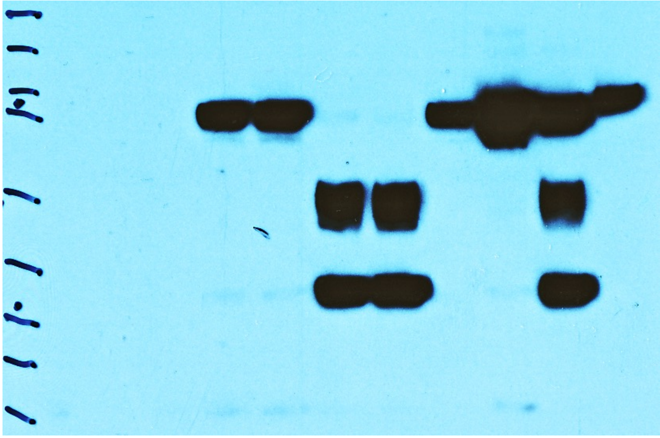

lanes used for the figure, right side, Non-reduced condition

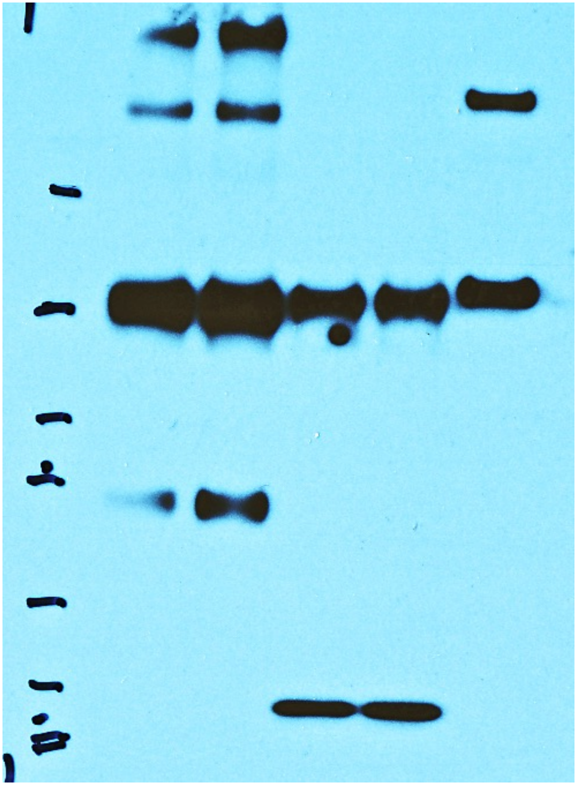

Full unedited membranse for Figure 3E, pAKT and AKT

lanes used for the figure, pAKT Ab,
the lanes were run on the same gel but were noncontiguous

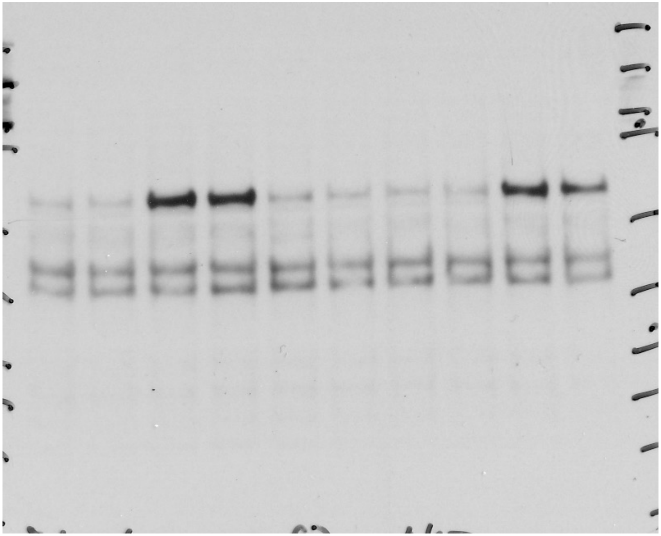

lanes used for the figure, AKT Ab,
the lanes were run on the same gel but were noncontiguous

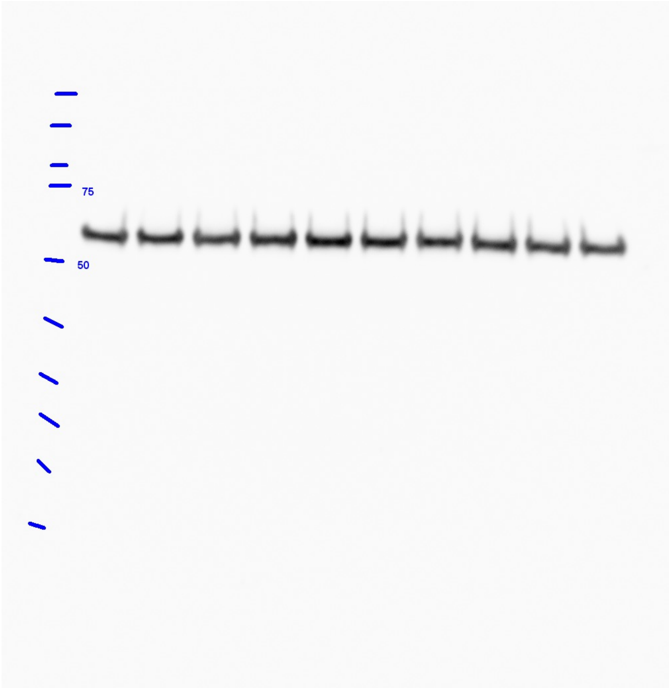

Full unedited film for Figure 3F

lanes used for the figure, pAKT

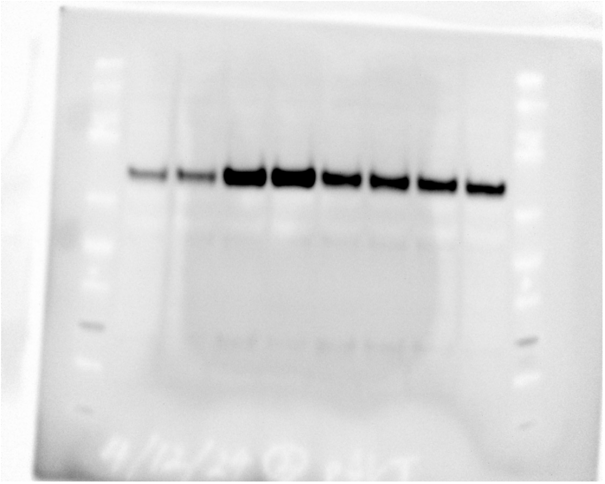

lanes used for the figure, AKT

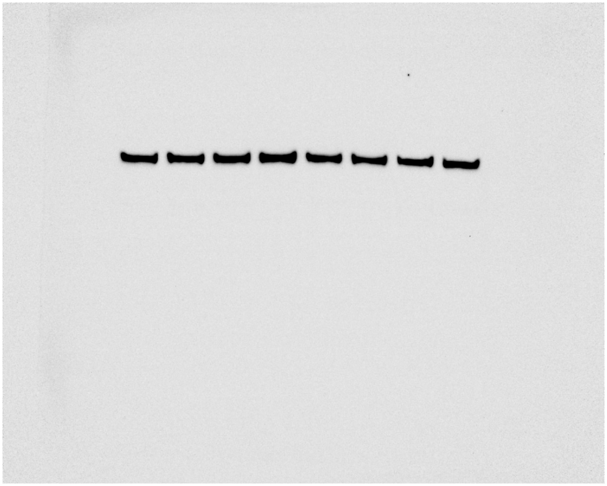

Full unedited membrane for Figure 6A

lanes used for the figure

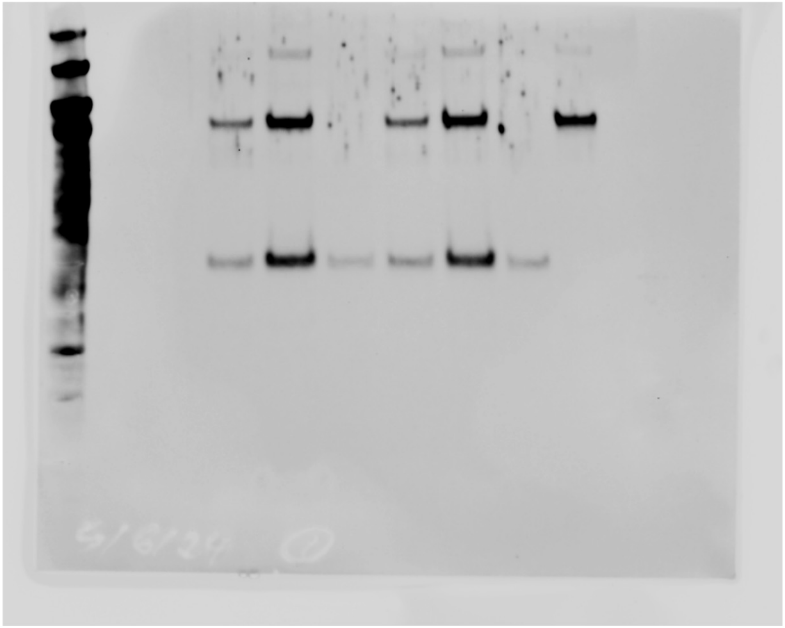

Full unedited membranes for Supplemental Figure 1B

lanes used for the figure, ANGPT2

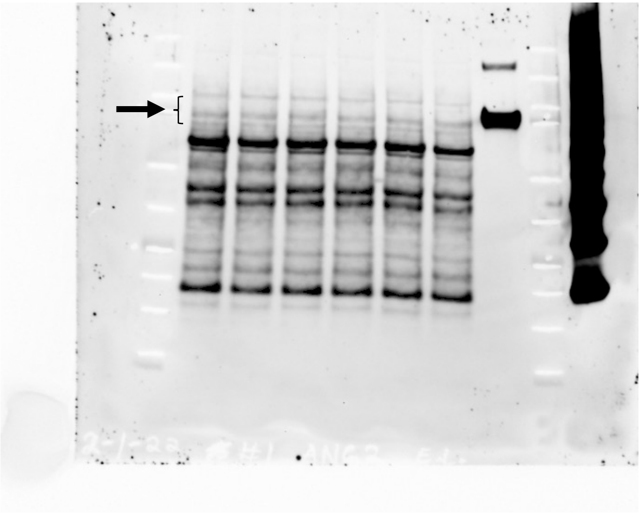

lanes used for the figure, ACTB

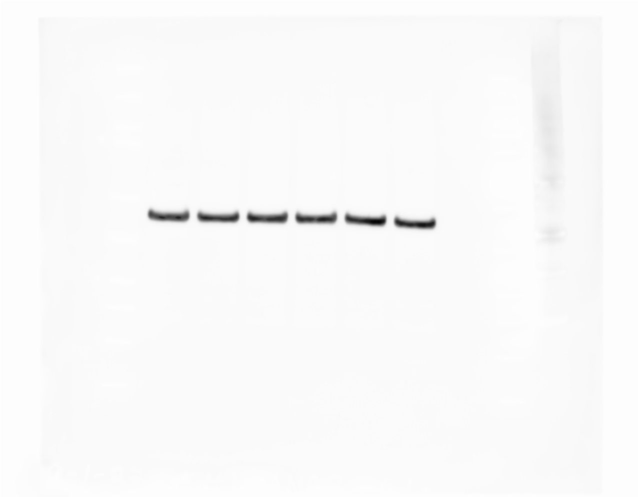

Full unedited membranes for Supplemental Figure 1C

lanes used for the figure, Tie2

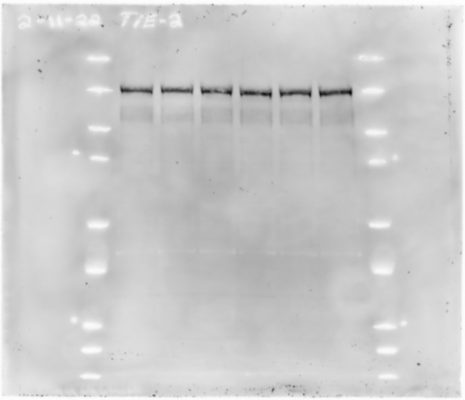

lanes used for the figure, ACTB

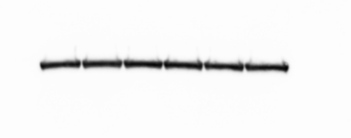

Full unedited membrane for Supplemental Figure 1D

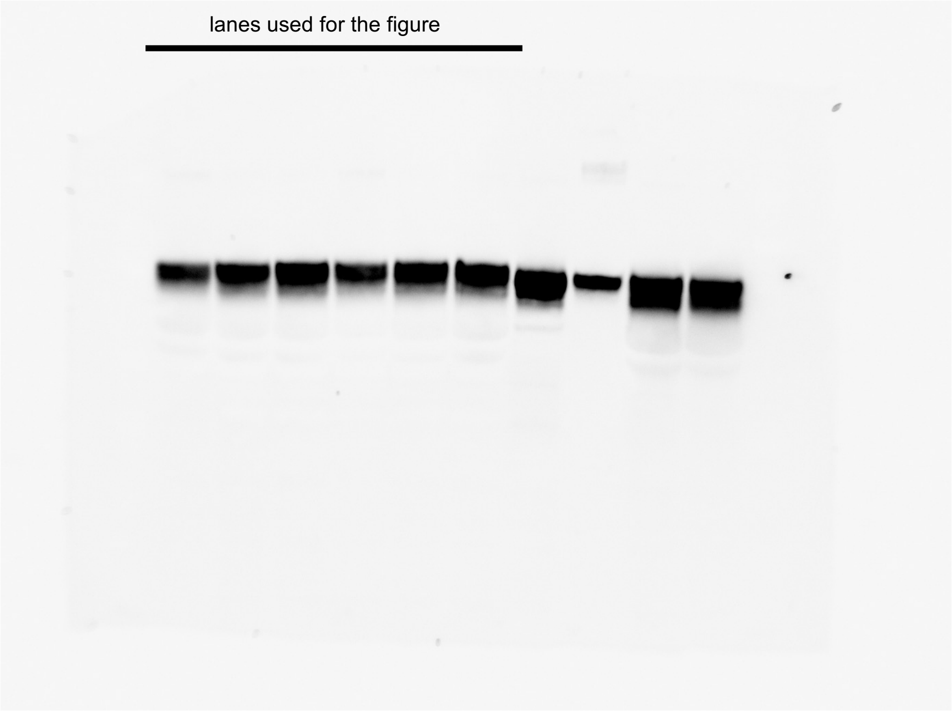

Full unedited membrane for Supplemental Figure 1E

lanes used for the figure

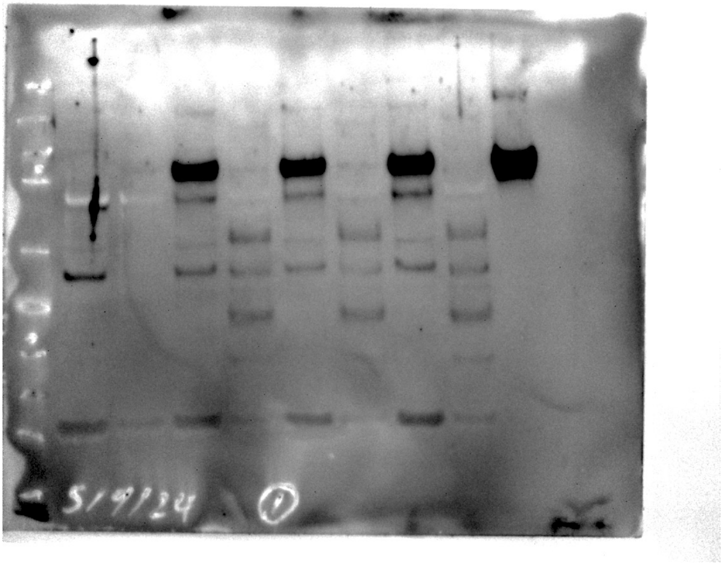

Full unedited membrane for Supplemental Figure2A

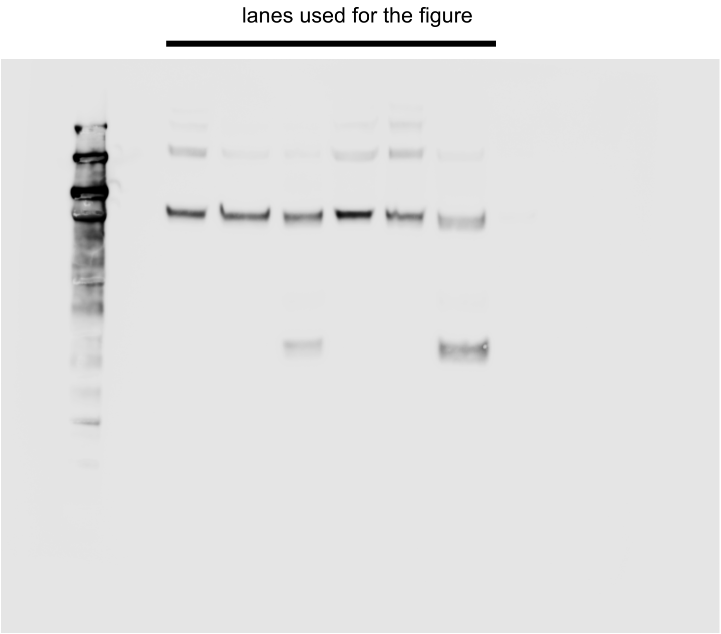

Full unedited membrane for Supplemental Figure2B

lanes used for the figure

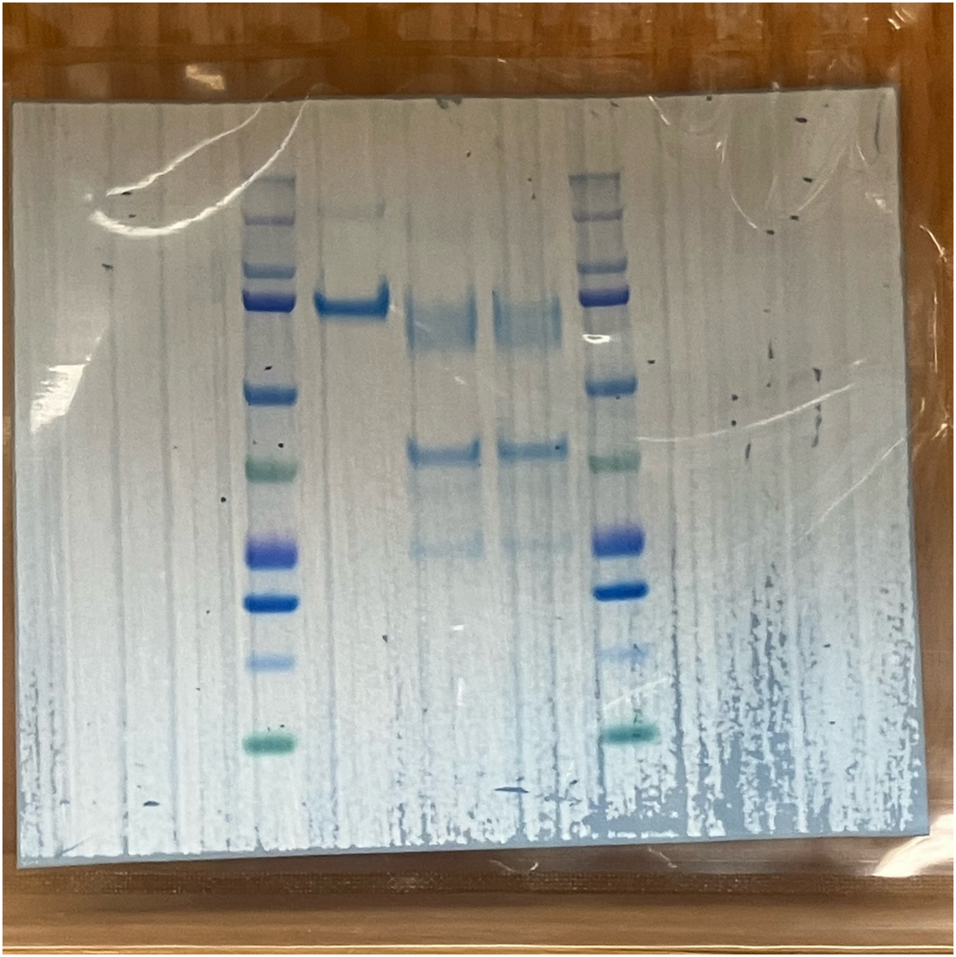

Full unedited membrane for Supplemental Figure2C

lanes used for the figure

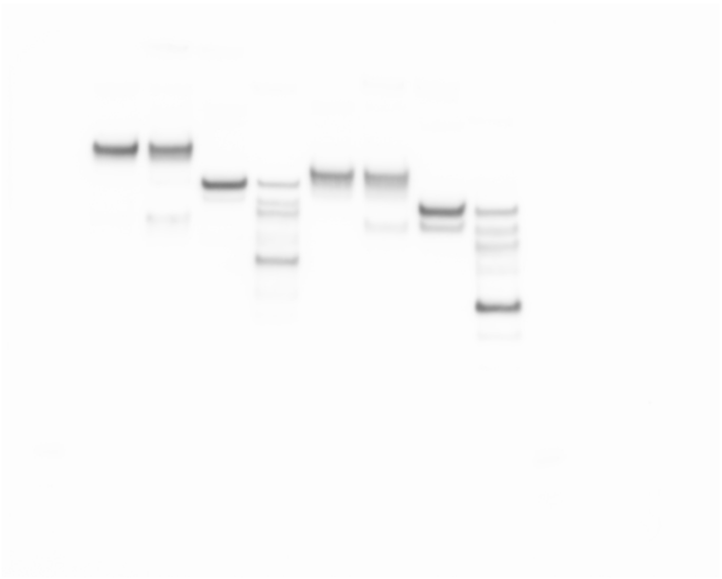

Full unedited membrane for Supplemental Figure 3

lanes used for the figure, sTie1

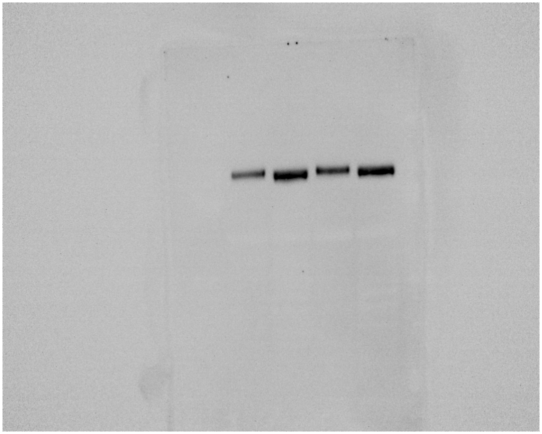

lanes used for the figure, sTie2

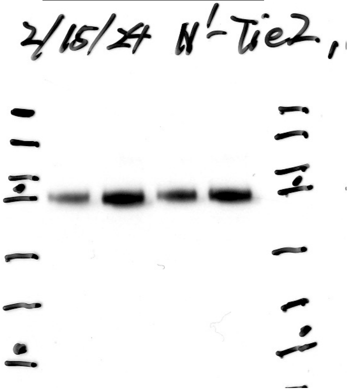

Full unedited membrane for Supplemental Figure 5

lane used for the figure, CM-Mq^{LPS} + rANGPT2

lanes used for the figure, pulldown

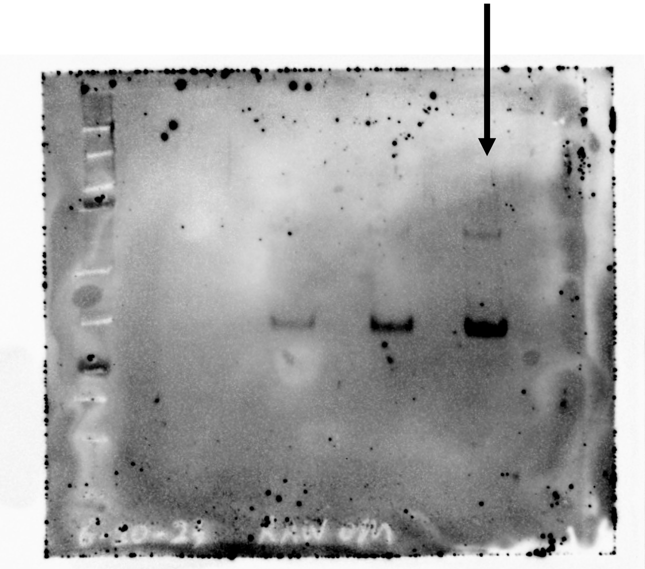

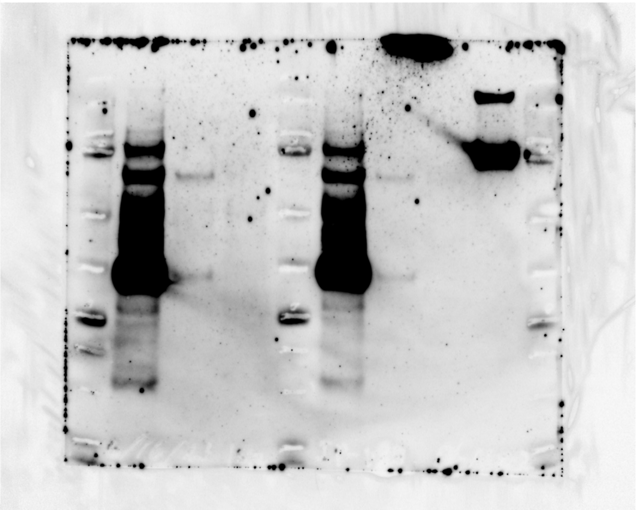

Full unedited membrane for Supplemental Figure 6G

lanes used for the figure

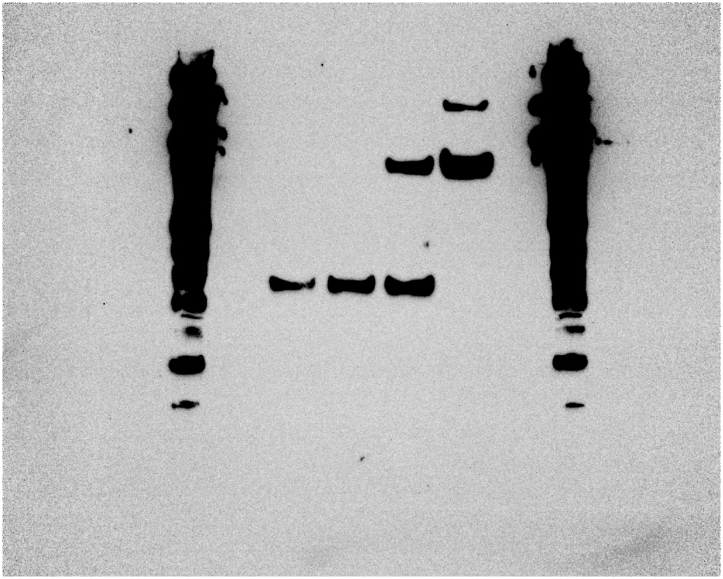

Full unedited membrane for Supplemental Figure 6J

lanes used for the figure, C'-ANGPT2

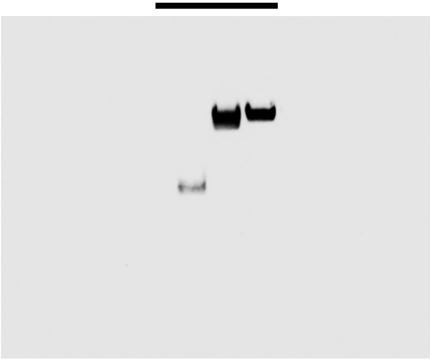

This Figure is also used in Figure 2D

lanes used for the figure, ANGPT2 (404-432)

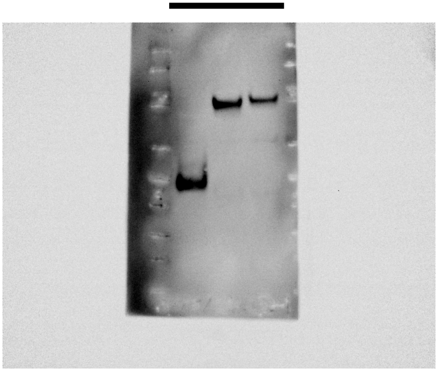

lanes used for the figure, His-tag

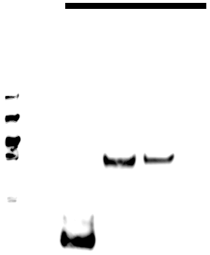

Full unedited membrane for Supplemental Figure 6K, L

lanes used for the figure, His-tag

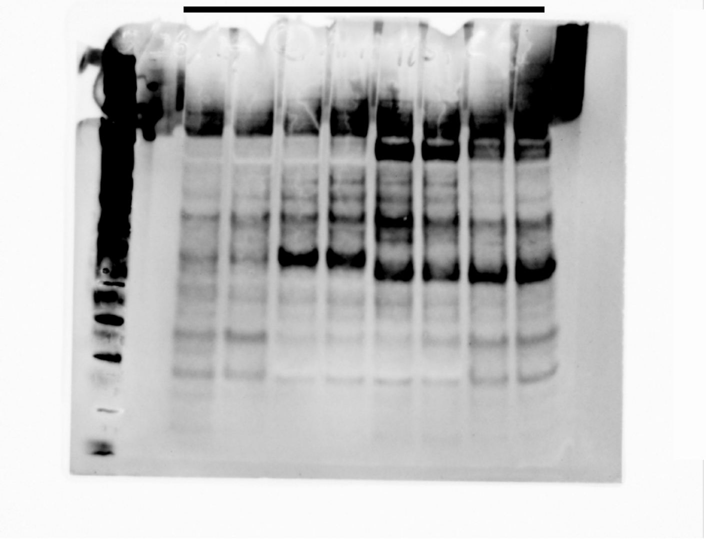

lanes used for the figure, β-actin

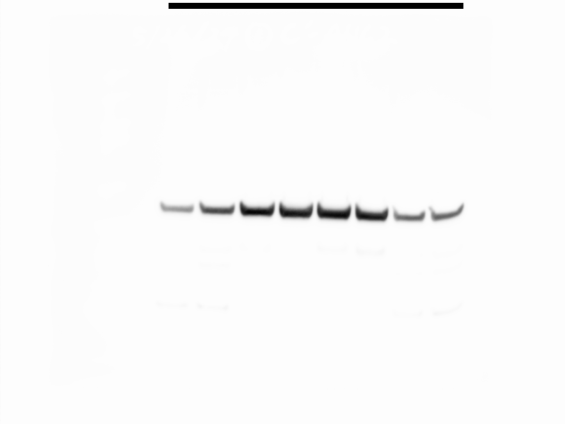

Full unedited membrane for Supplemental Figure 6N

lanes used for the figure

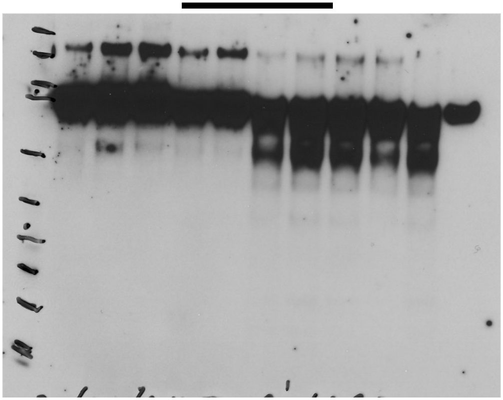

Full unedited membrane for Supplemental Figure 9

lanes used for the figure

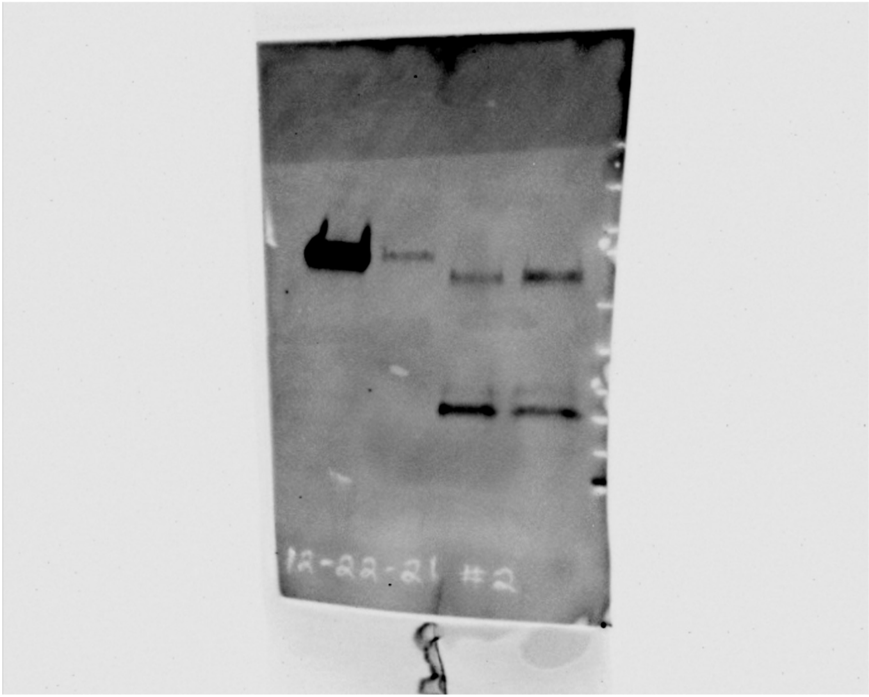

Full unedited membrane for Supplemental Figure 10

lanes used for the figure

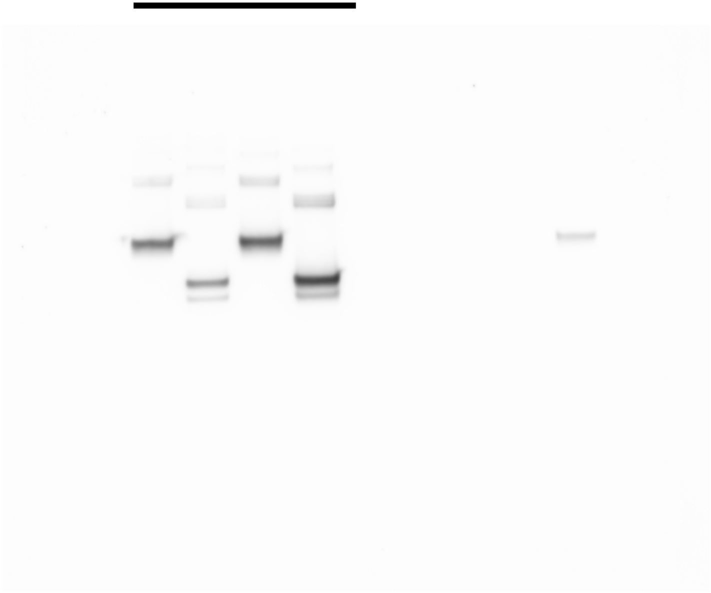

Supplement: Unedited blot and gel images [file jci-135-174135-s310.pdf]
